# E. coli Toxin YjjJ (HipH) Is a Ser/Thr Protein Kinase That Impacts Cell Division, Carbon Metabolism, and Ribosome Assembly

**DOI:** 10.1128/msystems.01043-22

**Published:** 2022-12-20

**Authors:** Fabio Lino Gratani, Till Englert, Payal Nashier, Peter Sass, Laura Czech, Niels Neumann, Sofia Doello, Petra Mann, Rudolf Blobelt, Siegfried Alberti, Karl Forchhammer, Gert Bange, Katharina Höfer, Boris Macek

**Affiliations:** a Quantitative Proteomics, Interfaculty Institute of Cell Biology, University of Tübingen, Tübingen, Germany; b Microbial Bioactive Compounds, Interfaculty Institute for Microbiology and Infection Medicine, University of Tübingen, Tübingen, Germany; c SYNMIKRO Research Center & Faculty of Chemistry, University of Marburg, Marburg, Germany; d Organismic Interactions, Interfaculty Institute for Microbiology and Infection Medicine, University of Tübingen, Tübingen, Germany; e Max Planck Institute for Terrestrial Microbiology, Marburg, Germany; NIAID, NIH

**Keywords:** cell division, kinases, metabolism, phosphoproteomics, proteomics, serine/threonine kinases

## Abstract

Protein Ser/Thr kinases are posttranslational regulators of key molecular processes in bacteria, such as cell division and antibiotic tolerance. Here, we characterize the E. coli toxin YjjJ (HipH), a putative protein kinase annotated as a member of the family of HipA-like Ser/Thr kinases, which are involved in antibiotic tolerance. Using SILAC-based phosphoproteomics we provide experimental evidence that YjjJ is a Ser/Thr protein kinase and its primary protein substrates are the ribosomal protein RpmE (L31) and the carbon storage regulator CsrA. YjjJ activity impacts ribosome assembly, cell division, and central carbon metabolism but it does not increase antibiotic tolerance as does its homologue HipA. Intriguingly, overproduction of YjjJ and its kinase-deficient variant can activate HipA and other kinases, pointing to a cross talk between Ser/Thr kinases in E. coli.

**IMPORTANCE** Adaptation to growth condition is the key for bacterial survival, and protein phosphorylation is one of the strategies adopted to transduce extracellular signal in physiological response. In a previous work, we identified YjjJ, a putative kinase, as target of the persistence-related HipA kinase. Here, we performed the characterization of this putative kinase, complementing phenotypical analysis with SILAC-based phosphoproteomics and proteomics. We provide the first experimental evidence that YjjJ is a Ser/Thr protein kinase, having as primary protein substrates the ribosomal protein RpmE (L31) and the carbon storage regulator CsrA. We show that overproduction of YjjJ has a major influence on bacterial physiology, impacting DNA segregation, cell division, glycogen production, and ribosome assembly.

## INTRODUCTION

Bacteria can quickly adapt to different growth conditions, which allows them to face continuous environmental changes and proliferate in numerous ecological niches. Sensing and responding to intra- and extracellular stimuli entails the regulation of many essential cellular mechanisms. The ability to rapidly and efficiently convert different signals into physiological response is the cornerstone of bacterial survival and adaptability (1). Dynamic protein phosphorylation is one of the key strategies used by the cell to transduce and convert the extracellular signals in the correspondent cellular response (2). The most known and studied examples in bacteria are the two-component systems (TCS), composed of a membrane bound receptor histidine kinase and a response regulator, generally a transcription regulator (3). Besides TCS, Ser/Thr/Tyr kinases represent another phosphorylation-based strategy for signal transduction and posttranslational regulation (4, 5). Contrary to TCS, members of this family of kinases typically phosphorylate multiple targets, affecting multiple aspects of cell physiology. Targets of Ser/Thr/Tyr kinases range from transcriptional and translational regulators (including TCS regulators), to metabolic enzymes as well as stress response proteins, underlying the crucial role of this regulatory mechanism in bacterial physiology (6). Recent studies demonstrate that Ser/Thr/Tyr phosphorylation is also involved in the regulation of antibiotic tolerance and persistence (7). The prominent example is the HipA kinase, a member of the *hipBA* Toxin-Antitoxin system (TA system), composed by the toxin HipA, which inhibits growth, and an antitoxin HipB, which counteracts the toxin activity (8). During normal growth conditions, the two genes are coexpressed and HipA is inactive; however, under specific conditions HipB is degraded, leading to HipA activation and growth inhibition. In this state of low metabolic activity, some bacterial cells can survive antibiotic treatments (antibiotic tolerance).

The kinase HipA inhibits growth by phosphorylating several proteins that are involved in different biological processes. A well-studied target of HipA is the glutamate-tRNA ligase GltX (7, 9). Phosphorylation by HipA inhibits the action of GltX and leads to an increase in the concentration of uncharged tRNA, mimicking amino-acid starvation and inducing growth inhibition. In a previous study we investigated the targets of HipA and its variant HipA7, which is responsible for high incidence of persister cells (10). Among the identified targets of HipA7 was the protein YjjJ, a toxin recently classified as a member of the family of HipA-like kinases and termed HipH (11, 12). This classification was supported by sequence similarity with HipA and conservation of amino acid residues involved in ATP and magnesium coordination, which are strictly required for kinase activity (12–15). However, unlike *hipA*, the *yjjJ* gene is not located in an operon with an antitoxin and its kinase activity was so far not experimentally verified.

Here, we show that YjjJ is a protein kinase that phosphorylates and negatively regulates the ribosomal protein RpmE (L31) and carbon storage regulator CsrA. Unlike HipA, overproduction of YjjJ does not directly lead to antibiotic tolerance but negatively impacts cell division and DNA segregation, ribosome assembly and regulation of central carbon metabolism. Overexpression of YjjJ influences the activity of HipA and other Ser/Thr kinases, and the resulting cell toxicity can be rescued by coexpression of the antitoxin HipB, pointing to a cross talk between these important regulatory proteins in bacterial cells.

## RESULTS

### YjjJ overproduction inhibits growth but has no direct impact on antibiotic tolerance.

**FIG 1:**
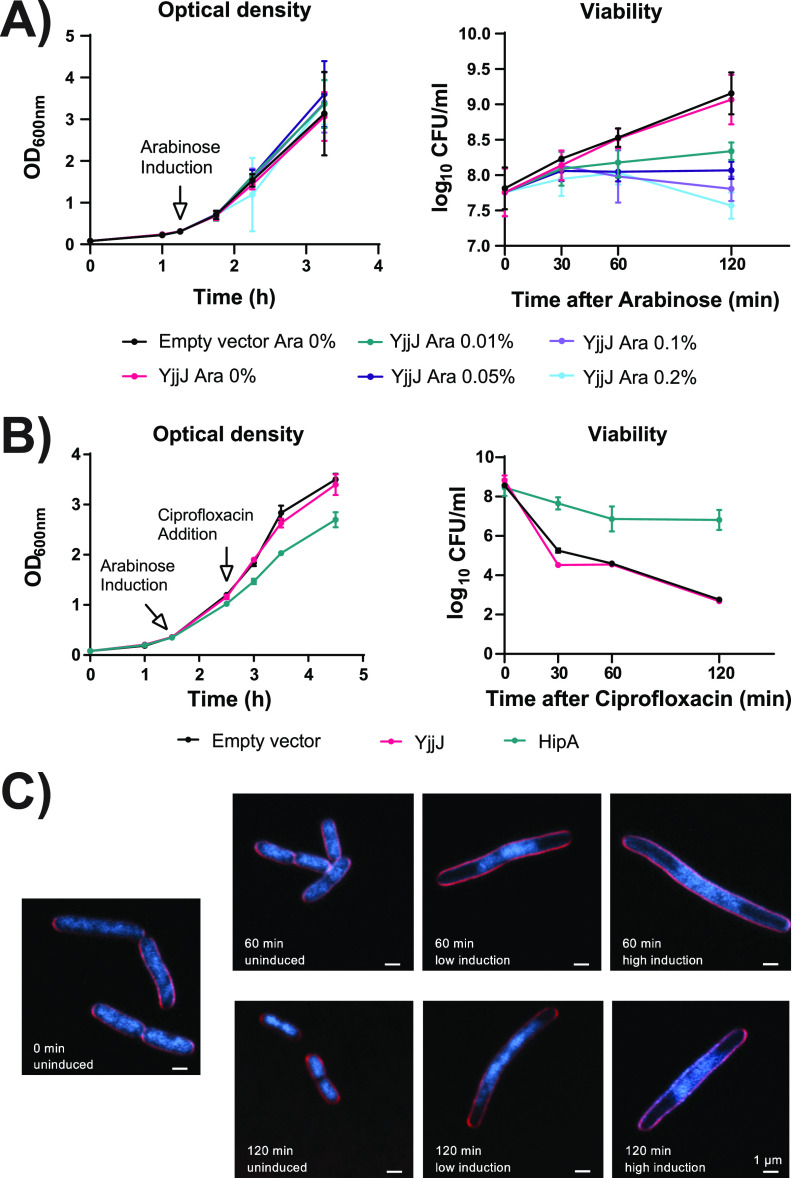
Ectopic expression of *yjjJ* leads to cell death, does not influence antibiotic tolerance, and impacts cell division and DNA segregation. (A) Growth curves of MG1655 strains transformed with either empty pBAD33 (Empty vector) or pBAD33::*yjjJ* (YjjJ) plasmid, in which *yjjJ* expression was under the control of arabinose-inducible promoter. Strains were grown in LB medium and expression was induced at OD_600_ of 0.3 using different arabinose concentrations. Growth was followed via optical density (OD_600_) and CFU (CFU). (B) Antibiotic tolerance of MG1655 strains transformed with empty vector, pBAD33::*yjjJ* (YjjJ) or pBAD33::*hipA* (HipA), in which *yjjJ* and *hipA* expression was driven by the arabinose-inducible promoter. Strains were grown in LB medium and expression was induced at OD_600_ of 0.3 for 1 h followed by 1 μg/mL ciprofloxacin. Growth was followed via optical density and CFU. (C) Superresolution microscopy of cells transformed with pBAD33::*yjjJ* in exponentially growing culture supplemented with 0.01% (low induction) or 0.2% (high induction) arabinose, compared to uninduced (control) cells. Cells were stained with FM5-95 (membrane, red) and DAPI (DNA, blue) and examined by superresolution fluorescence microscopy. Overlaid fluorescence images show membranes (red) and DNA (blue). Images were taken at the indicated time points (60 and 120 min) after the addition of arabinose. YjjJ overproduction resulted in filamentous cells, indicating inhibition of cell division. DAPI staining revealed a deficient nucleoid segregation and DNA degradation. Scale bar: 1 μm. Images are representative of at least two biological replicate cultures.

To investigate the function of *yjjJ*, we first assessed its impact on E. coli growth in LB medium. It was previously shown that strong overproduction of YjjJ leads to a drop in CFU (CFU) counts (11); therefore, we ectopically expressed *yjjJ* under the control of an inducible promoter and tested different concentrations of the inducer (arabinose) in order to identify the conditions that inhibited cell growth. Interestingly, we did not observe any significant difference between the tested conditions at the absorbance (OD_600_) level; however, an increase in induction strength significantly reduced the number of CFU in a dose-dependent manner (Fig. 1A). Two hours after induction, at any arabinose concentration tested, bacteria lost the plasmid in a manner proportional to the induction intensity, indicating that *yjjJ* expression was toxic for the cell (Fig S1A). Importantly, overexpression of *hipB* antitoxin rescued the toxicity of YjjJ overproduction (Fig S1B), as previously shown (11).

The significant homology of YjjJ to HipA suggested a similar role in antibiotic tolerance (7, 10, 16). Thus, we probed the impact of YjjJ overproduction on cells grown in the presence of either ampicillin or ciprofloxacin. Cells expressing *yjjJ* died at similar rates to those expressing the empty vector under both antibiotic treatments, which was in stark contrast to cells expressing *hipA* (Fig. 1B, Fig S1C). Therefore, we conclude that overproduction of YjjJ is toxic for the cell and does not impact antibiotic tolerance under the tested conditions.

### YjjJ overproduction impacts DNA segregation and cell division.

We next investigated whether YjjJ overproduction impacts subcellular structures, such as the cell membranes or the nucleoid. (Fig. 1C, Fig. S1D). In contrast to uninduced control cells, which showed regular chromosome segregation and cell division, induction of *yjjJ* resulted in a diffuse distribution of nucleoids accompanied by DNA degradation. As a consequence, division septa failed to form while cell length increased, resulting in filamentous cells up to seven times longer than uninduced cells. This filamentation phenotype was in agreement with the drop in CFU counts upon *yjjJ* induction, and it explained the steady increase of optical density observed in Fig. 1A (17). We conclude that overproduction of YjjJ has a strong negative impact on cell division and DNA segregation even at low induction conditions.

10.1128/msystems.01043-22.1 FIG S1 Ectopic expression of *yjjJ* results toxic, can be complemented by HipB, does not affect antibiotic tolerance and impact on cell division and DNA segragation. (A) Loss of plasmid after induction of YjjJ with different concentration of arabinose. Cells were streaked on LB and LB-chloramphenicol plates and percentage was calculated as survivor on selection plates over plain LB agar plates. (B) HipB complementation of YjjJ. *hipB* was cloned under the control of an IPTG-inducible promoter while *yjjJ* expression was under an arabinose-inducible promoter. MG1655 strains carrying pEG220 and pBAD33 (Empty vectors), pEG220 and pBAD33::*yjjJ* (Empty vector + YjjJ) or pEG220::*hipB* and pBAD33::*yjjJ* (HipB + YjjJ) were grown on LB to OD_600_ of 0.3 followed by addition of 0.1 mM IPTG and 0.1% arabinose. (C) Antibiotic tolerance toward ampicillin of MG1655 strains bearing empty vector, pBAD33::*yjjJ* (YjjJ) or pBAD33::*hipA* (HipA). Genes *yjjJ* and *hipA* are under control of an arabinose-inducible promoter. Strains were grown in LB and plasmid expression was induced at OD_600_ of 0.3, with 0.01% for YjjJ expressing cells and 0.2% arabinose for empty vector and HipA expressing cells for 1 h, followed by treatment with 100 μg/mL ampicillin. Cells were then harvested at different time points. Growth was followed via optical density and colony forming units. (D) Micrographs of cells before, one hour and two hours after arabinose induction as in Fig. 1. Scale bar: 5 μm. Download FIG S1, PDF file, 0.3 MB. Copyright © 2022 Gratani et al. 2022 Gratani et al. https://creativecommons.org/licenses/by/4.0/ This content is distributed under the terms of the Creative Commons Attribution 4.0 International license.

### Prolonged overproduction of YjjJ impacts carbon storage.

**FIG 2:**
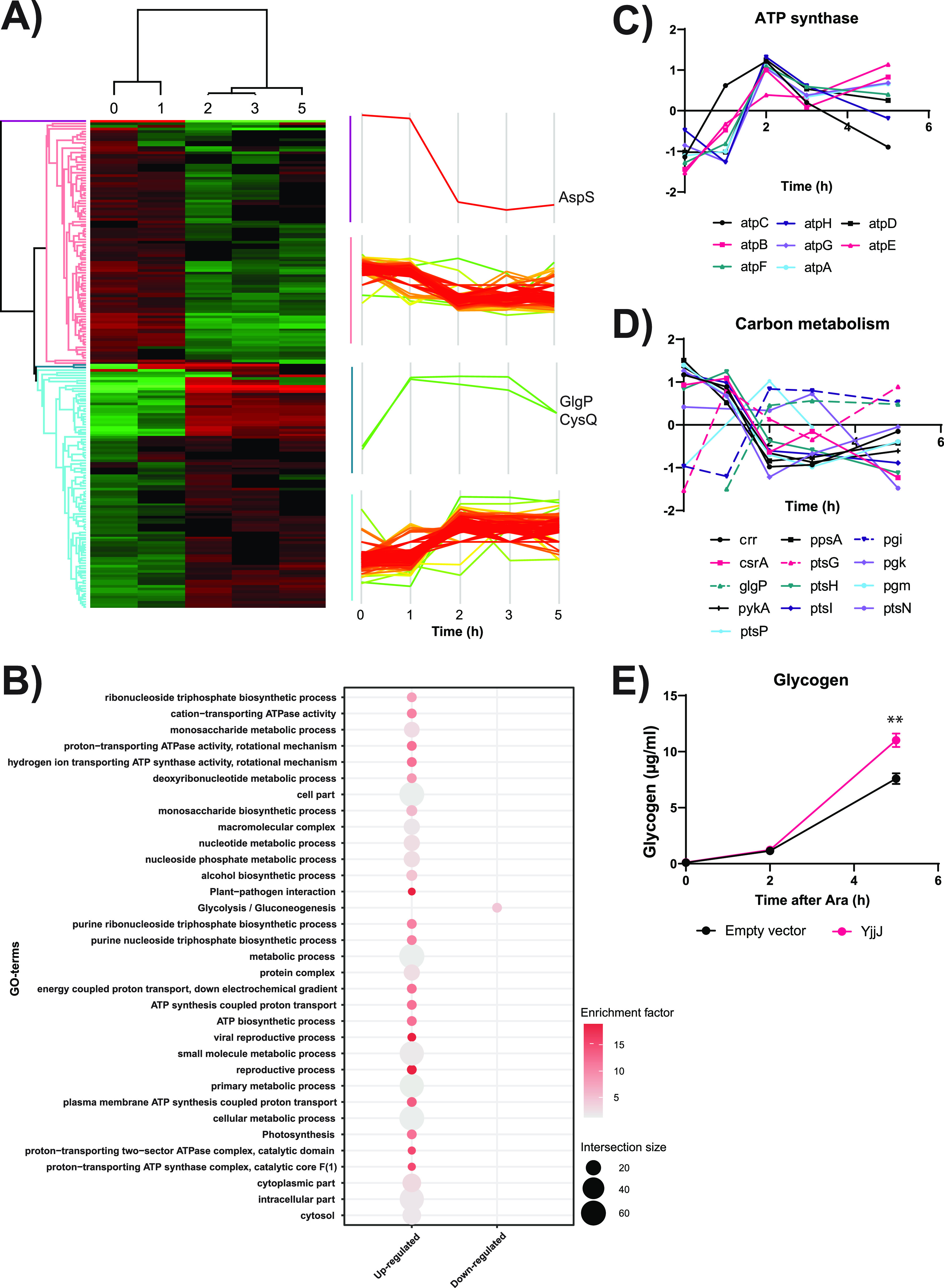
Prolonged overproduction of YjjJ impacts ATP synthesis and carbon storage. (A) Heat map of significantly regulated proteins after prolonged overproduction of YjjJ (Anova, FDR < 0.1; Post Hoc, FDR < 0.1). Color coding is based on YjjJ/Empty vector ratio. Temporal profiles were clustered on their dynamic over time. (B) Gene ontology (GO) enrichment of proteins grouped within the clusters. (C) Temporal profiles of selected proteins involved in ATP synthesis after induction of *yjjJ*. (D) Temporal profiles of selected proteins involved in carbon metabolism after induction of *yjjJ*. (E) Glycogen levels were measured in cells transformed with pBAD33 (Empty vector) or pBAD33::*yjjJ* (YjjJ), in which *yjjJ* expression is driven by the arabinose-inducible promoter. Cells were grown in LB medium until OD_600_ equal to 0.3 and supplemented with 0.01% arabinose. Bacteria were harvested before, two and 5 h after induction for absolute quantification of glycogen.

To further analyze YjjJ impact on the proteome dynamics, we ectopically induced *yjjJ* expression at low inducer concentration (i.e., 0.01% arabinose) for 5 h in LB, which led to slow growth and high YjjJ abundance compared to cells carrying the empty vector (Fig. S2A and B). In a MS-based quantitative proteomics measurement, we quantified 1,270 proteins in at least two replicates (Data set S1, Sheet 1). Significantly regulated proteins were clustered in four groups based on their temporal profiles (Fig. 2A). The two main clusters presented opposite trends: one increased over time and showed an enrichment in proteins related to ATP biosynthesis, such as ATP-synthase components, whereas the other decreased over time and was enriched in proteins related to carbon metabolism, such as glycolysis and gluconeogenesis (Fig. 2B to D, Fig. S2C). Interestingly, several tRNA-ligases were decreased in abundance, with a notable exception of LysU that strongly increased 2 h after YjjJ overproduction (Fig. S2D). The levels of RecA were increased, suggesting DNA damage upon YjjJ overproduction. MinD and HtpG, both related to cell division/elongation, changed in abundance compared to empty vector strain, providing a possible link between YjjJ and the observed filamentation phenotype (Fig. S2D) (18, 19). Two smaller clusters contained the tRNA ligase AspS, which decreased strongly already 1 h after *yjjJ* induction, as well as the glycogen phosphorylase GlgP and bisphosphate nucleotidase CysQ, which increased after *yjjJ* induction. Since proteomic analysis indicated that YjjJ overproduction influences central carbon metabolism, we next analyzed glycogen production in cells expressing *yjjJ*-plasmid or the empty vector. Five hours after mild *yjjJ* induction (i.e., 0.01% arabinose) we measured a significantly higher abundance of glycogen in cells overproducing YjjJ, compared to empty vectors cells (Fig. 2E, Fig. S2E). We therefore conclude that YjjJ overproduction impacts central carbon metabolism and storage.

10.1128/msystems.01043-22.2 FIG S2 Prolonged overproduction of YjjJ impacts growth and several physiological pathways. (A) Growth curves of MG1655 strains carrying either empty pBAD33 (Empty vector) or pBAD33::*yjjJ* (YjjJ), in which *yjjJ* expression was under the control of an arabinose-inducible promoter. Strains were grown in LB and expression was induced at OD_600_ of 0.3, with 0.01% arabinose. Growth was followed via optical density and CFU measurements. (B) Relative abundance of YjjJ during growth, as log_2_ ratio between cells bearing empty vector and YjjJ-expressing plasmid. (C) Gene ontology (GO) enrichment of proteins based on change of abundance among different time points. (D) Profiles (log_2_ ratio YjjJ/Empty vector) over time of selected proteins, as example of proteome dynamics. (E) Absolute quantification of protein amount and glycogen normalized to the protein content after arabinose induction (0.01%). Download FIG S2, PDF file, 0.7 MB. Copyright © 2022 Gratani et al. 2022 Gratani et al. https://creativecommons.org/licenses/by/4.0/ This content is distributed under the terms of the Creative Commons Attribution 4.0 International license.

**FIG 3:**
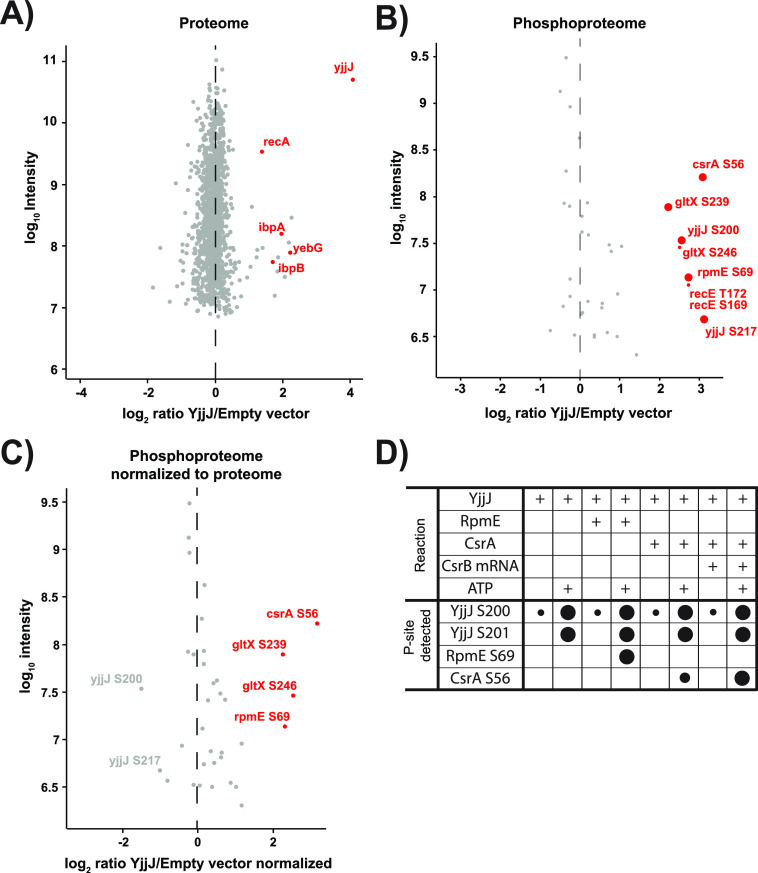
YjjJ is a protein kinase that phosphorylates CsrA, RpmE, and itself. (A) SILAC ratios of proteins measured 2 h after *yjjJ* induction. (B) Representation of phosphorylated sites following normalization to proteome abundance. The names of the phosphorylated proteins and the positions of the phosphorylation sites showing at least a 4-fold change in phosphorylation (red) are indicated. (C) Distribution of phosphorylation site ratios after normalization to the corresponding protein levels. The regulated sites (4-fold change in phosphorylation) are marked in red. (D) *In vitro* kinase assay of YjjJ with RpmE and CsrA as the substrates. After the phosphorylation reaction, the samples were analyzed by LC-MS/MS. Large circles: increased phosphorylation at the indicated sites was detected in at least two independent experiments. Small circles: two orders-of-magnitude lower intensity of phosphorylation site on YjjJ without ATP addition to the reaction mix; for CsrA the small circle represents an almost order of magnitude lower signal than without mRNA. Results represent an average of at least two replicates.

10.1128/msystems.01043-22.10 DATA SET S1 Sheet 0: Column description for Sheet 1–10, Sheet 1: Dimethyl label - Proteome dynamics protein groups (Fig. 2, Fig. S2), Sheet 2: SILAC-based - protein groups (Fig. 3, Fig. S3), Sheet 3: SILAC-based - phosphorylation sites (Fig. 3, Fig. S3), Sheet 4: SILAC-based, YjjJ^DK^ native background - protein groups (Fig. S4), Sheet 5: SILAC-based, YjjJ^DK^ native background - phosphorylation sites (Fig. S4), Sheet 6: SILAC-based, YjjJ^DK^ *ΔhipBA* background protein groups (Fig. S4), Sheet 7: SILAC-based, YjjJ^DK^ *ΔhipBA* background - phosphorylation sites (Fig. S4), Sheet 8: *In vitro* kinase - protein groups (Fig. 3, Fig. S5), Sheet 9: *In vitro* kinase - phosphorylation sites (Fig. 3, Fig. S5), Sheet 10: Proteome crude ribosome extract (Fig. S6). Download Data Set S1, XLSX file, 4.2 MB. Copyright © 2022 Gratani et al. 2022 Gratani et al. https://creativecommons.org/licenses/by/4.0/ This content is distributed under the terms of the Creative Commons Attribution 4.0 International license.

10.1128/msystems.01043-22.3 FIG S3 YjjJ impacts proteome and phosphoproteome with good reproducibility. (A) Growth curves of E. coli K-12 MG1655 carrying the pBAD33*::yjjJ* plasmid (YjjJ), in which *yjjJ* expression is under the control of an arabinose-inducible promoter, or pBAD33 as empty vector control. Strains were grown in SILAC-labelled minimal medium containing stable isotope labelled lysine derivatives: “light” lysine (Lys0), “medium-heavy” lysine (Lys4), or “heavy” lysine (Lys8). (B) Correlation of proteins and phosphorylation-site SILAC ratios from the *yjjJ* expressing strain relative to the strain bearing the empty vector for the three independent replicates. (C) Volcano plot representing distributions of quantified proteins amongst the three replicates. Proteins with a significant increase (FDR < 0.1) are marked in red. (D) Gene ontology (GO) distribution of those proteins showing an increase in abundance 2 h after *yjjJ* expression, enriched against the background of all identified proteins. Download FIG S3, PDF file, 0.8 MB. Copyright © 2022 Gratani et al. 2022 Gratani et al. https://creativecommons.org/licenses/by/4.0/ This content is distributed under the terms of the Creative Commons Attribution 4.0 International license.

10.1128/msystems.01043-22.4 FIG S4 YjjJ induction cross-talks with HipA pathways, leading to phosphorylation of GltX. (A) Growth curves of E. coli K-12 MG1655 wild type carrying the pBAD33*::yjjJ* or pBAD33*::yjjJ S342,364Q* (YjjJ^DK^) plasmid, in which gene expression is under the control of an arabinose-inducible promoter, or pBAD33 as empty vector control. Strains were grown in LB medium. After reaching OD_600_ of 0.3, plasmid expression was induced with 0.2% arabinose. Growth was followed at OD_600_ and CFU level. (B) Quantified proteins represented as log_2_ ratio between YjjJ native and YjjJ^DK^ expressing cells. Significantly changing proteins (*P* < 0.05) are indicated (red) with good correlations between the two replicates. (C) Distribution of phosphorylation sites upon YjjJ overproduction based on log_2_ ratio between native YjjJ and YjjJ^DK^ expressing cells shows good correlation between the two replicates. (D) Growth curve of E. coli Δ*hipBA* carrying either pBAD33*::yjjJ* or pBAD33*::yjjJ S342,364Q* (YjjJ^DK^) plasmid. Growth was followed at OD_600_ and CFU level. (E) Quantified proteins represented as log_2_ ratio between native YjjJ and YjjJ^DK^ expressing cells in Δ*hipBA* background. Significantly changing proteins (*P* < 0.05) are indicated (red) with good correlations between the two replicates. (F) Distribution of phosphorylated sites upon YjjJ overproduction in the Δ*hipBA* background, based on log_2_ ratio between native YjjJ and YjjJ^DK^ expressing cells shows good correlation between the two replicates. Download FIG S4, PDF file, 1.0 MB. Copyright © 2022 Gratani et al. 2022 Gratani et al. https://creativecommons.org/licenses/by/4.0/ This content is distributed under the terms of the Creative Commons Attribution 4.0 International license.

10.1128/msystems.01043-22.5 FIG S5 CsrA phosphorylation is confirmed by *in vitro* kinase autoradiography, indicating mRNA as cofactor. (A) LysS complementation of YjjJ. *lysS* was cloned under the control of an IPTG-inducible promoter while *yjjJ* expression was driven by the arabinose-inducible promoter. MG1655 strains carrying pEG50 and pBAD33 (Empty vectors), pEG220 and pBAD33::*yjjJ* (Empty vector + YjjJ) or pEG25::*lysS* and pBAD33::*yjjJ* (LysS + YjjJ) were grown on LB to OD_600_ of 0.3, followed by addition of 1 mM IPTG and 0.1% arabinose. (B) *In vitro* phosphorylation of CsrA. Purified YjjJ was incubated with [γ^32P^] ATP, in the presence or absence of CsrC mRNA. The figure depicts the gel was Coomassie stained, radiograms of the gels and the merge. The negative control is represented by CsrA S56,59E mutant. (C) Control reactions of *in vitro* kinase assay, measured by mass spectrometry, of Fig. 2. Download FIG S5, PDF file, 2.8 MB. Copyright © 2022 Gratani et al. 2022 Gratani et al. https://creativecommons.org/licenses/by/4.0/ This content is distributed under the terms of the Creative Commons Attribution 4.0 International license.

**FIG 4:**
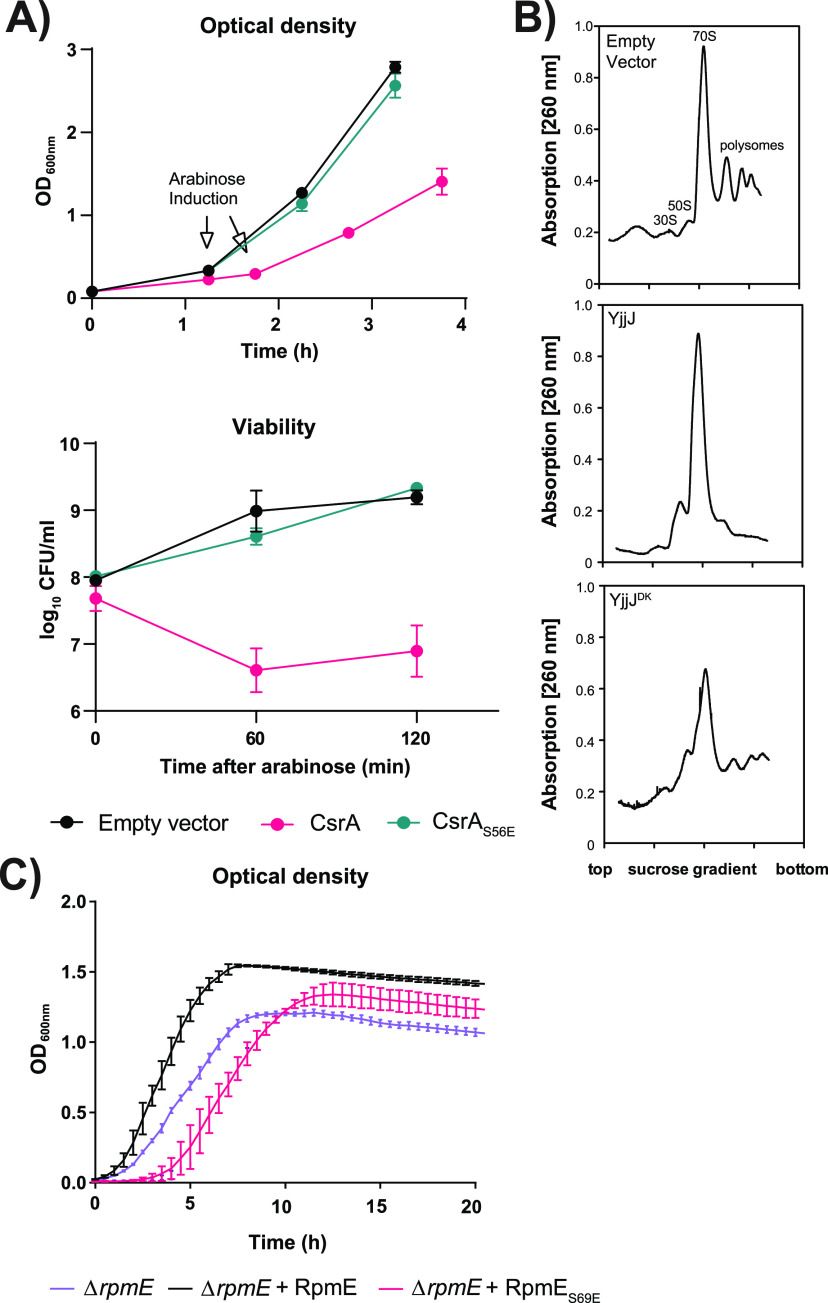
YjjJ kinase negatively regulates the function of carbon storage regulator CsrA and ribosomal protein RpmE (L31). (A) Impact of CsrA and phosphomimetic mutant CsrA_S56E_ on E. coli viability. Growth curves of MG1655 strains carrying either empty pBAD33 (Empty vector), pBAD33::*csrA* (CsrA) or pBAD33::*csrA_S56E_* (CsrA_S56E_), in which gene expression was under the control of an arabinose-inducible promoter. Cells were grown until OD_600_ of 0.3 and induced with 0.2% arabinose. Growth was followed at OD_600_ and CFU level. (B) Effect of YjjJ on ribosome assembly. Cells bearing pBAD33 (Empty vector), pBAD33::*yjjJ* or pBAD33::*yjjJ S342*,*364Q* (YjjJ^DK^) were induced during exponential growth with 0.01% arabinose until OD_600_ of 0.7, followed by harvest. Ribosomes were extracted and separated on a sucrose gradient. (C) Complementation of RpmE and phosphomimetic mutant RpmE_S69E_ on E. coli Δ*rpmE*. Cells carrying either pEG25::*rpmE* (RpmE) or pEG25::*rpmE_S69E_* (RpmE_S69E_) were grown until an OD_600_ of 0.1 and induced with 1 mM IPTG. Growth was followed via OD_600_ measurements.

10.1128/msystems.01043-22.6 FIG S6 Control experiments confirm toxicity of YjjJ and its interaction with ribosome. (A) Electromobility assay of CsrA. Different amounts of native and phosphomimetic (S56,59E) CsrA were incubated with CsrC mRNA to test its interaction. F represents unbound and B represents bound mRNA. (B) Second replicate of ribosome assembly profile from Fig. 4C. (C) MS analysis of crude ribosome extract from ribosome assembly profile experiment. Distributions of proteins represented as log_2_ ratio between YjjJ^DK^/Empty vector and YjjJ/Empty. Protein presenting 4-fold change of abundance are indicated (red). (D) Growth comparison between MG1655 wild type and Δ*rpmE* as control for Fig. 4C. Download FIG S6, PDF file, 0.3 MB. Copyright © 2022 Gratani et al. 2022 Gratani et al. https://creativecommons.org/licenses/by/4.0/ This content is distributed under the terms of the Creative Commons Attribution 4.0 International license.

### YjjJ is a protein kinase that phosphorylates CsrA, RpmE, and itself.

YjjJ was proposed to be a protein kinase (11), but experimental evidence of its function was so far missing. To investigate *in vivo* YjjJ kinase activity and identify its putative targets, we used a SILAC-based phosphoproteomics approach, as described previously (10). Briefly, we induced overexpression of *yjjJ* in E. coli cultured in minimal medium supplemented with stable isotope-labeled derivatives of lysine (Lys0 and Lys8), and performed phosphoproteome analysis using liquid chromatography coupled to tandem mass spectrometry (LC-MS/MS). We compared two strains: one with the empty vector (Lys0 label) and one with the *yjjJ*-expressing plasmid (Lys8 label). After bacteria reached an OD_600_ of 0.4, we induced *yjjJ* expression for 2 h (Fig. S3A). At the protein level, we measured a strong increase in YjjJ abundance in triplicate measurements, confirming the efficiency of the expression strategy (Fig. 3A, Fig. S3B to D, Data set S1, Sheet 2). At the phosphoproteome level, we identified 201 phosphorylation sites on 126 proteins with good correlation among replicate measurements (Fig. S3B, Data set S1, Sheet 3). Upon YjjJ overproduction, we reproducibly detected an increase in the phosphorylation of the glutamate tRNA-ligase GltX (Ser239), carbon storage regulator CsrA (Ser56, Ser59) and L31 ribosomal protein RpmE (Ser69), making these proteins putative targets of YjjJ (Fig. 3B and C). In addition, we detected (auto)phosphorylation sites on YjjJ itself (Ser200, Ser201 and Ser217). Several additional phosphorylation sites were upregulated but could not be normalized for protein levels and will not be discussed further (Data set S1, Sheet 3).

### Overproduction of kinase-dead YjjJ (YjjJ^DK^) reveals an interplay between HipA and other kinases.

Since overproduction of YjjJ may potentially activate other Ser/Thr kinases and bias the phosphoproteomics results, we repeated the SILAC phosphoproteome screen using a kinase-dead mutant of YjjJ (YjjJ^DK^). Importantly, mutations in the putative Mg^2+^- and ATP-binding domains diminished the toxic effect on cell growth (Fig. S4A and D). On the phosphoproteome level, phosphorylation of most of the identified targets (CsrA, RpmE and YjjJ autophosphorylation) did not increase during YjjJ^DK^ overproduction, confirming them as putative YjjJ targets. However, phosphorylation levels on GltX were similar in YjjJ- and YjjJ^DK^-overproducing cells, indicating that GltX phosphorylation cannot be attributed to YjjJ activity alone (Fig. S4A to C, Data set S1, Sheet 4,5).

Since our previous study indicated potential cross talk between HipA and YjjJ activity (10), we reasoned that increased GltX phosphorylation may have resulted from HipA activation in the YjjJ-overproducing E. coli strain. To address this, we overproduced YjjJ or the YjjJ^DK^ mutant in *ΔhipBA* background. Under these conditions we could not detect an increase in GltX phosphorylation (Fig. S4D to F; Data set S1, Sheet 6 and 7), revealing that the observed GltX phosphorylation after *yjjJ* induction should be attributed to the HipA kinase. Intriguingly, overproduction of YjjJ^DK^ also led to a significant increase in the phosphorylation of the lysine tRNA-ligase LysS at Thr133 (Fig. S4C and F). This increase was not driven by a change in protein levels of LysS, and was also detected in the *ΔhipBA* background, revealing that LysS is phosphorylated neither by HipA nor by YjjJ. We therefore conclude that YjjJ^DK^ overproduction indirectly regulates LysS phosphorylation, likely by regulation of a yet unknown kinase or a phosphatase. Of note, overexpression of *lysS* could not complement the toxic effect of *yjjJ* overexpression (Fig. S5A), and the exact physiological connection between the two genes remains to be determined.

To validate potential YjjJ substrates from the SILAC screen, we performed *in vitro* kinase assays with purified YjjJ and confirmed direct phosphorylation of RpmE and CsrA (Fig. 3D, Fig. S5B and C, Data set S1, Sheet 8 and 9). Interestingly, *in vitro* phosphorylation of CsrA was only observed when the small CsrB RNA was added to the assay, revealing that the RNA-bound pool of CsrA is predominantly targeted by YjjJ. Taken together, SILAC-based and *in vitro* phosphoproteomics experiments showed that YjjJ is a protein kinase that directly phosphorylates CsrA, RpmE and itself.

### YjjJ kinase impacts activity of the carbon storage regulator CsrA.

To further explore the influence of YjjJ on CsrA function, we overproduced either CsrA_WT_ or its phospho-mimetic version CsrA_S56E_ in wild type E. coli and followed the growth over time. Overexpression of *csrA_wt_* led to a drop in CFU by an order of magnitude, compared to uninduced cells. Importantly, unlike CsrA_WT_, overexpression of CsrA_S56E_ did not affect cell growth (Fig. 4A), indicating that YjjJ-mediated phosphorylation negatively regulates CsrA. Interestingly, the interaction of CsrA_S56E_ with CsrC RNA did not change in electrophoretic mobility shift assays (Fig. S6A), indicating that YjjJ phosphorylation does not affect the interaction of CsrA with its RNA targets. This is in agreement with the *in vitro* kinase assay results showing that YjjJ preferentially phosphorylates RNA-bound CsrA. Taken together, these results demonstrate that YjjJ-mediated phosphorylation on Ser56 negatively affects the function of CsrA.

### YjjJ overproduction affects RpmE (L31) function and ribosome assembly.

The YjjJ substrate RpmE (L31) is a small ribosomal protein positioned at the interface between the 30S and the 50S ribosomal subunits (20). It was previously reported that deletion of eight amino acids at the C terminus of RpmE impairs the correct assembly of the 70S ribosome (20). Since the phosphorylated Ser69 is located in the C-terminal region of RpmE, we postulated that YjjJ-mediated phosphorylation may impact the ribosome assembly. To address this, we first analyzed ribosome profiles after moderate overproduction (i.e., 0.01% arabinose) of either native YjjJ or YjjJ^DK^ and compared it to the strain expressing empty plasmid (Fig. 4B, Fig. S6B). Ribosomes purified from cells bearing empty plasmid showed the typical ribosomal profile with peaks representing 30S, 50S, 70S, and polysomes (Fig. 4B). Interestingly, overproduction of YjjJ led to disappearance of the 30S- and polysome signals and an increase in the 50S signal. Overproduction of YjjJ^DK^ only mildly altered the native ribosome assembly and the elution profile, indicating that YjjJ impacts the ribosome assembly but not as the sole regulator. Interestingly, MS analysis showed the presence of YjjJ (both native and kinase-dead) in crude ribosome extracts, indicating the interaction between YjjJ and the ribosome (Fig. S6C, Data set S1, Sheet 10). To determine the potential phenotypic effect of RpmE (L31) phosphorylation, we performed a complementation experiment in the Δ*rpmE* strain, which is characterized by a delayed entrance in the exponential phase. Complementation of the *rpmE* null mutant with native *rpmE* rescued bacterial growth, while the phosphomimetic version *rpmE_S69E_* further delayed the entrance into exponential phase, demonstrating that YjjJ-mediated phosphorylation at Ser69 negatively regulates RpmE activity (Fig. 4C, Fig. S6D). In summary, these results demonstrate that YjjJ overproduction impacts ribosome assembly by phosphorylation and negatively affects the function of RpmE.

## DISCUSSION

Bacterial Ser/Thr protein kinases HipA and YjjJ both belong to the HipA-kinase superfamily and contain the same conserved motifs (Mg- and ATP-binding sites) and an autophosphorylation site (16). Homologs of YjjJ are spread among different bacterial families, indicating that this kinase is likely involved in conserved mechanism(s) of bacterial physiology (Fig. S7). Here, we confirm that strong ectopic overexpression of *yjjJ* leads to cell death, showing the need for a tight regulation of its endogenous levels. Overproduction of YjjJ at lower levels slows the growth in batch culture, which is a phenotype often connected to antibiotic tolerance (21). However, in contrast to HipA-overproducing cells, YjjJ-overproducing cells are almost equally sensitive to ampicillin and/or ciprofloxacin treatment as control cells. Therefore, under the investigated conditions YjjJ is not directly involved in the establishment or maintenance of antibiotic tolerance. Nevertheless, several indirect lines of evidence connect the function of YjjJ and HipA: (i) the high persistence variant HipA7 was shown to phosphorylate YjjJ *in vivo* (10); (ii) the antitoxin HipB can rescue the toxic effect of both, HipA and YjjJ ([10] and this study); (iii) overproduction of YjjJ affects the activity of HipA (this study). The latter is demonstrated by increased levels of GltX phosphorylation in *yjjJ*-expressing cells, which cannot be attributed to YjjJ action. We speculate that the two kinases are likely connected at two levels: directly (via kinase activity) and indirectly (via competition for HipB). For example, HipA-mediated GltX phosphorylation in cells overproducing kinase-dead YjjJ (YjjJ^DK^) may be explained by YjjJ^DK^-mediated sequestering of HipB, which in turn increases the pool of active HipA copies. However, an exact understanding of the mechanistic and functional aspects of the cross talk between the two kinases will require a dedicated study.

10.1128/msystems.01043-22.7 FIG S7 (A) Distribution of *yjjJ* among different genera (upper panel) and percentage of identity (lower panel). (B) Multiple sequence alignment (Clustal Omega) of 16 different YjjJ, 13 HipA, and three ¾ kinase protein sequences from various organisms. (C) Visualization of motifs identified in multiple sequence alignment. (D) Pairwise sequence alignment between YjjJ and HipA proteins. Download FIG S7, PDF file, 1.5 MB. Copyright © 2022 Gratani et al. 2022 Gratani et al. https://creativecommons.org/licenses/by/4.0/ This content is distributed under the terms of the Creative Commons Attribution 4.0 International license.

YjjJ overproduction resulted in cell elongation and aberrant DNA segregation during cell division. This abnormal phenotype explained the increase of optical density of *yjjJ*-expressing cell cultures over time, despite the decrease in CFU. Upon induction of *yjjJ* we identified changing levels of HtpG and MinCDE, previously shown to influence cell shape and division (18, 19), which link YjjJ action to the observed phenotypes in cell division and DNA segregation. We also identified a strong increase of RecA over time, which indicates DNA stress and strengthen the potential damaging effect of YjjJ on DNA.

Careful tuning of the *yjjJ* expression levels allowed us to identify nontoxic inducing conditions and to analyze the physiological effect of YjjJ on the proteome level over a longer period of time. YjjJ overproduction caused an increase in the levels of ATP-related proteins and a decrease in the levels of proteins related to glycolysis and gluconeogenesis, indicating a rearrangement in energy production similar to that observed at the entrance of the stationary phase (22). This was further supported by overall downregulation of proteins involved in sugar transport and metabolism, as well as protein biosynthesis (e.g., tRNA-ligases). Despite the drop in CFU, pellet from *yjjJ*-expressing cells had a similar glycogen content as pellet from cells bearing the empty vector, which confirmed higher relative amounts of glycogen in *yjjJ*-expressing cells.

At the phosphoproteome level, overproduced YjjJ phosphorylates L31 (rpmE), CsrA, and itself. The phosphorylation site on RpmE is located in a region needed for correct ribosome assembly (20). Induction of *yjjJ* resulted in defects in ribosome assembly similar to deletion of *rpmE* (23), and overproduction of the kinase-dead mutant of YjjJ (YjjJ^DK^) partially recovered the defective ribosome assembly. The phosphomimetic mutant *rpmE*_S69E_ failed to complement the defective growth of the Δ*rpmE* strain, confirming that YjjJ-mediated phosphorylation negatively regulates RpmE. During stationary phase, in which a rearrangement of L31a (*rpmE*) and L31b (*ykgM*) takes place (24), the strong expression of native *rpmE* was toxic. We speculate that under these conditions L31a (*rpmE*) completely displaced L31b in the ribosome structure, impairing the correct ribosome rearrangement. This effect was not present in cells overexpressing the phosphomimetic *rpmE*_S69E_, suggesting that YjjJ activity could be related to a rearrangement of ribosomal proteins in the stationary phase.

YjjJ-mediated phosphorylation of the carbon storage regulator CsrA is positioned on its regulatory domain (25–27). We showed that the strong toxic effect of CsrA overproduction in E. coli is absent in cells overexpressing the phosphomimetic mutant CsrA_S56E_, demonstrating that YjjJ negatively regulates CsrA activity. The location of the phosphorylated Ser56 is distant from the mRNA-binding and dimerization domain of CsrA, but it is situated in a regulatory region (25–27). Interestingly, deletion of the *csrA* gene was previously shown to lead to higher glycogen content and longer cell shape (28, 29), phenotypes that we observed in YjjJ-overproducing cells.

Of note, deletion or CRISPRi-mediated silencing of the *yjjJ* gene did not have any significant effect on the cell growth (Fig. S8) or proteome dynamics (unpublished results) during exponential growth in batch culture, likely due to very low expression of the gene. However, most of the phenotypical consequences of YjjJ induction, such as arrest of cell division and DNA segregation, increased glycogen synthesis, and altered ribosome assembly, are hallmarks of the stationary phase physiology. In this context it is interesting that previous studies showed an increase in *yjjJ* transcript levels during mid- and late-exponential phase (30) as well as an increase in the YjjJ protein level at the beginning of the stationary phase (31). This suggests that the kinase YjjJ is likely involved in regulatory mechanisms that govern bacterial physiology in the stationary phase and future research should address this aspect of YjjJ biology.

10.1128/msystems.01043-22.8 FIG S8 Effect of CRISPRi-Silencing and Deletion of YjjJ on growth. (A) Growth curve of E. coli strain *YYdCas9:BW25993* cells with *yjjJ*-pgRNA. Expression of dCas9 induced by aTc 100ng/mL to repress *yjjJ* or not induced (as a control). Cells were grown in M9 minimal medium in 24-well microtiter plates. Growth was followed in two independent experiments. Samples harvested at the end of the growth curve for repression assay. (B) Repression assay for relative mRNA levels of YjjJ induced versus not induced by doing real-time PCR with cDNA. Data are normalized to the expression of the endogenous control gene, *hcaT*. (C) Growth curve of E. coli *K-12* MG1655 carrying the empty vector and E. coli *ΔyjjJ* carrying either the empty vector or pBAD33::*yjjJ* plasmid (YjjJ) in which *yjjJ* expression was under the control of an arabinose-inducible promoter. Strains were grown in LB medium and expression was induced at OD_600_ of 0.3, with 0.2% arabinose. Growth was followed via optical density and CFU measurements. (D) Table for confirmation of knockout. Strains were grown in SILAC-labelled minimal medium containing stable isotope labelled lysine derivatives: “light” lysine (Lys0), “medium-heavy” lysine (Lys4), or “heavy” lysine (Lys8), harvested after 2 hrs of induction with 0.2% arabinose, followed by sample preparation and measurement via MS. Download FIG S8, PDF file, 0.6 MB. Copyright © 2022 Gratani et al. 2022 Gratani et al. https://creativecommons.org/licenses/by/4.0/ This content is distributed under the terms of the Creative Commons Attribution 4.0 International license.

## MATERIALS AND METHODS

### Bacterial strains and plasmids.

Strains, plasmids, primers, and cloning strategies used in this work are listed in the Text S1.

10.1128/msystems.01043-22.9 TEXT S1 Supplementary Materials and Methods. This file contains extended description of Materials and Methods, including relevant references. Download Text S1, PDF file, 0.3 MB. Copyright © 2022 Gratani et al. 2022 Gratani et al. https://creativecommons.org/licenses/by/4.0/ This content is distributed under the terms of the Creative Commons Attribution 4.0 International license.

### Bioinformatic analysis.

PsiBLAST analysis was performed at the NCBI server (https://blast.ncbi.nlm.nih.gov/Blast.cgi). The sequence analysis of YjjJ focused on the motifs found in the protein, following the pipeline described in the Text S1.

### Growth experiments.

Culturing conditions and different growth strategies are described in the Text S1.

### SILAC labeling.

For quantitative phosphoproteomic experiments, E. coli cells were differentially labeled using stable isotope–labeled lysine derivatives as described previously (32). For more details, please refer to the Text S1.

### Dimethyl-labeling.

For the quantitative measurement of proteome dynamics, digested samples were on-stage tip dimethylation labeled as described previously (33), as explained in the Text S1.

### Cell lysis and protein extraction.

For proteomic analysis, proteins were extracted following the protocol explained in the Text S1.

### Protein digestion in solution.

Extracted proteins were digested for LC-MS/MS measurements. Different digestion strategies are detailed in the Text S1.

### Phosphopeptide enrichment.

For phosphoproteome experiment, previous LC-MS/MS measurement, phosphopeptides were enriched following the protocol described in the Text S1.

### Incorporation and mixing check.

The efficiency of SILAC labeling was determined by LC-MS/MS measurement of Lys4- and Lys8-labeled samples, as well as for dimethyl labeling. The different protocols are explained in the Text S1.

### Peptide purification by StageTips.

Before each LC-MS/MS measurement, all peptide samples were desalted and purified on C18 StageTips (34), as described in the Text S1.

### LC-MS/MS measurement.

Purified peptide samples were separated by an EASY-nLC 1000 or 1200 system (Thermo Fisher Scientific) coupled online to a Q Exactive HF mass spectrometer (Thermo Fisher Scientific) through a nanoelectrospray ion source (Thermo Fisher Scientific). Chromatographic separation was performed on a 20-cm-long, 75–μm–inner diameter analytical column packed in-house with reversed-phase ReproSilPur C18-AQ 1.9 μm particles (Maisch GmbH). The column temperature was maintained at 40°C using an integrated column oven. The different strategies adopted for the different experiment are detailed in the Text S1.

### MS data processing and analysis.

The different processing and data analysis pipelines for the different experiments are explained in details in the Text S1.

### Protein purification.

Plasmids for protein purification were transformed in E. coli One Shot BL21(DE3) and the different purification protocols are described in the Text S1.

### *In vitro* transcription of CsrC.

DNA template for *csrC* transcripts was amplified by PCR. PCR products were analyzed by 1% agarose gel electrophoresis and purified using the QIAquick PCR purification kit (Qiagen). CsrC-RNA was synthesized by *in vitro* transcription (IVT) in the presence of 40 mM Tris, pH 8.1, 1 mM spermidine, 10 mM MgCl_2_, 0.01% (Triton X-100), 5% DMSO, 10 mM DTT, 4 mM each NTP, 20 μg of T7 RNA polymerase (2 mg/mL) and 200 nM DNA template. For the preparation of 32-P-labeled csrC transcripts, IVT was performed in the presence of 0.4 μCi/μL ^32^P-α-ATP (Hartmann Analytics). The IVT reaction mixtures were incubated at 37°C for 4 h and digested with DNase I (Roche). RNA was purified by denaturing PAGE and isopropanol-precipitation, resuspended in Millipore water, and RNA concentration determined by NanoDrop measurements.

### Electromobility shift assay.

EMSA was performed to study the interaction of CsrA or CsrA S56,59E to *crsC* transcripts. The binding assay was conducted in the presence of 0.6 nM radioactively labeled csrC RNA, 10 mM Tris–HCl, pH 7.5, 10 mM MgCl_2_, 100 mM KCl, 7.5% glycerol, 20 mM DTT, 200 ng yeast tRNA and various concentrations (0 to 600 nM) of CsrA or CsrA S56,59E. Reaction mixtures were incubated at 30°C for 30 min and analyzed via 9% native polyacrylamide gels in precooled TBE buffer. Radiolabeled csrC transcripts were visualized using storage phosphor screens (GE Healthcare) and a Typhoon 9400 imager (GE Healthcare).

### Phosphorylation assay of CsrA by YjjJ for autoradiography.

Phosphorylation of CsrA and CsrA S56,59E was performed in the presence of 2 mM DTT, 10 mM MgCl_2_, 8 mM ZnCl_2_, 50 mM Tris pH 8.1, 0.5 mM ATP, 5 μCi ^32^P-γ-ATP (Hartmann Analytics), 5 μM YjjJ, and 15 μM CsrA or CsrA S56,59E for 1h at 37°C. To characterize the influence of RNA, 6 pmol of CsrC was added to the reaction. Samples were taken before the addition of YjjJ (0 min), after 60 min of incubation and stopped by the addition of Tricine Loading Dye each. The reactions were analyzed by 15% Tricine-SDS-PAGE, autoradiography imaging and Coomassie staining.

### *In vitro* kinase assay for MS analysis.

Kinase (1.2 μg) (His6-YjjJ) was incubated with 1.2 μg His-tagged substrate in a kinase buffer (50 mM Tris-HCl pH = 8,1 10 mM MgCl_2_, 16 μM [ZnCl2]) with or without 10 mM ATP and with or without 6 pmol of CsrC for CsrA. Each reaction contained 2.4 μg of a total protein amount. Samples were incubated at 37°C for 2 h and stopped by the addition of nine volumes of denaturation buffer. Samples were split, followed by the protein digestion using chymotrypsin or Lys-C endoproteinase, as previously described (see above). Digested peptides were purified using StageTips (see above), and 0.2 μg of each sample was measured by LC-MS/MS (see above).

### Super-resolution fluorescence microscopy.

E. coli were grown as described previously, at OD_600_ of 0.3 YjjJ expression was induced with 0.01% and 0.2% arabinose and cells were harvest at defined time points. Cells were stained using FM5-95 (10 μg/mL; Molecular Probes) and 4′,6-diamidino-1-phenylindole (DAPI; 1 μg/mL; Sigma-Aldrich) dyes for 7 min to visualize membranes and nucleoids, respectively. Then, samples were mounted on microscopy slides coated with 1% agarose in water to immobilize cells. Images were acquired using the Zeiss Axio Observer Z1 LSM800 equipped with Airyscan detector. Image analysis was performed via ZEN image analysis software (Zeiss).

### Ribosome purification and density gradient.

Ribosomes were purified and the assembly analyzed following the protocol in the Text S1.

### Glycogen measurement.

Glycogen was determined as described previously (35), with following modifications: 50 mL of culture were harvested and total absolute glycogen content was determined.

### Transduction of *rpmE* and *yjjJ* deletion.

Deletion strains of *rpmE* and *yjjJ*, in MG1655, were perpared by transduction with P1*vir* lysate, using the KEIO donor strain following the protocol explained in the Text S1.

### CRISPRi *yjjJ* repression assay.

Transformation of *YYdCas9*:BW25993 *intC::TetR-dcas9-aadA laqY::ypet-cat* (36) with the *yjjJ*-pgRNA plasmid (37) with the protocol described in the Text S1, followed by growth curve determination. RNA preparation for Real-time PCR to see the expression of *yjjJ* with details in the Text S1.

### Data availability.

The mass spectrometry proteomics data have been deposited to the ProteomeXchange Consortium via the PRIDE (38, 39) partner repository with the data set identifier PXD033071.
