# Supplementary material for: E. coli Toxin YjjJ (HipH) Is a Ser/Thr Protein Kinase That Impacts Cell Division, Carbon Metabolism, and Ribosome Assembly
Source: mSystems. 2022 Dec 20;8(1):e01043-22. doi: 10.1128/msystems.01043-22 (PMC9948734; doi:10.1128/msystems.01043-22)
Supplement: FIG S1 [file msystems.01043-22-s0002.pdf]

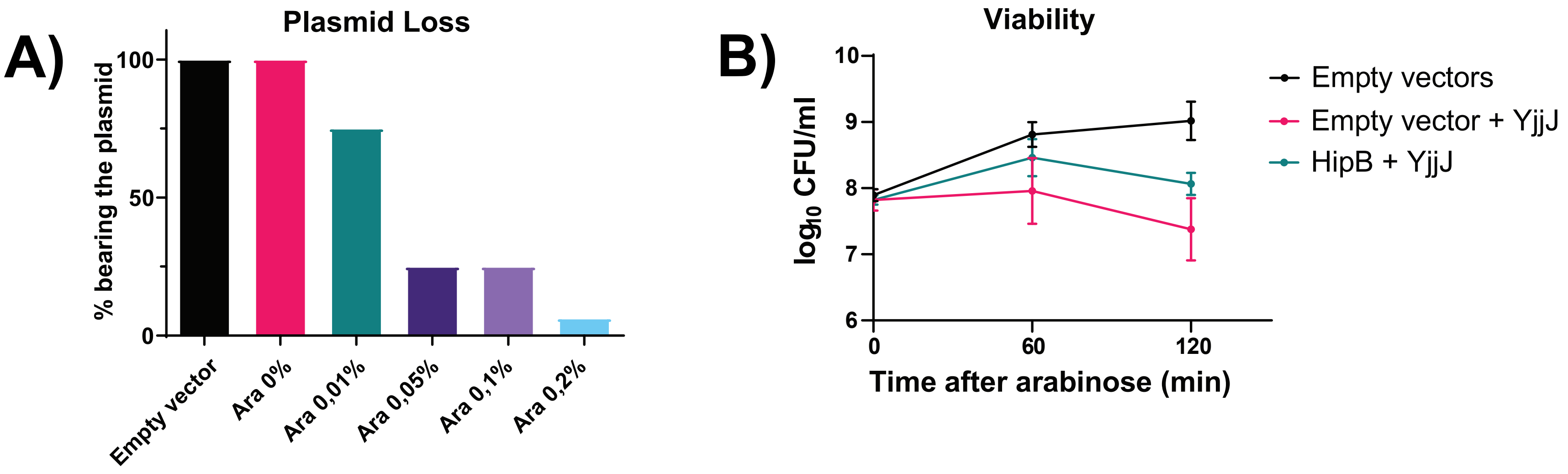

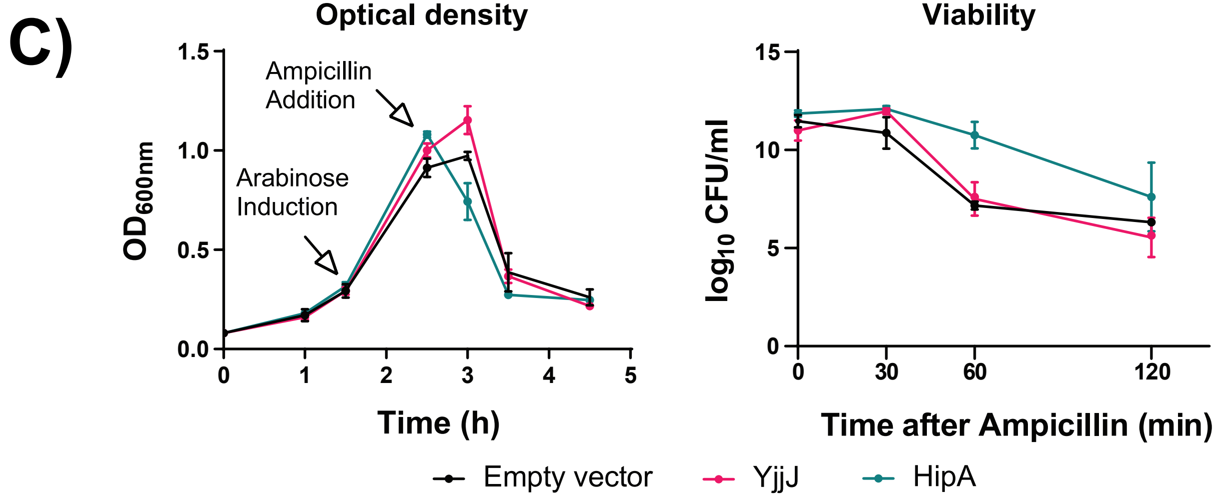

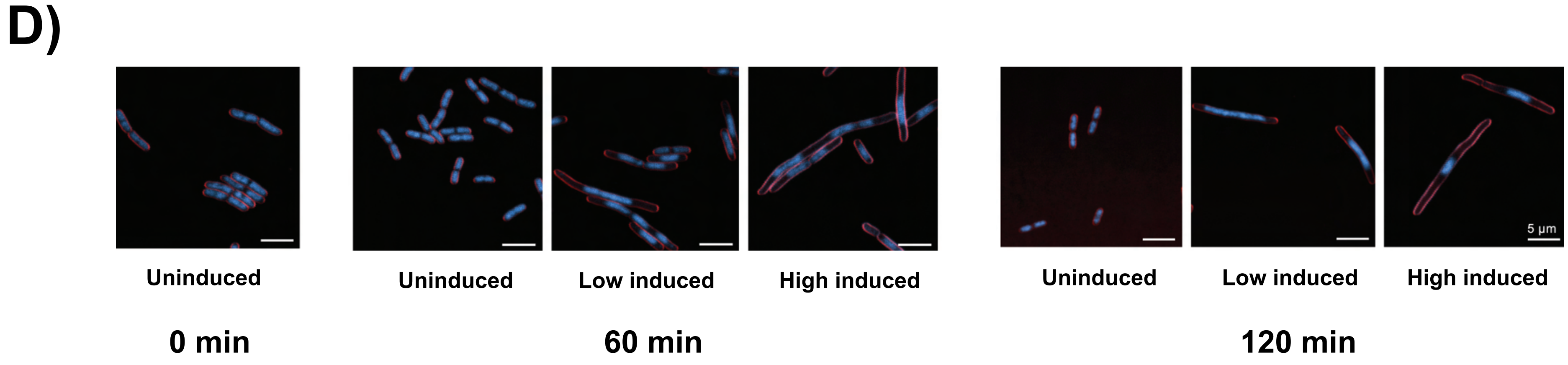

Fig.S1: Ectopic expression of *yjjJ* results toxic, can be complemented by HipB, doesn't affect antibiotic tolerance and impact on cell division and DNA segragation.
A) Loss of plasmid after induction of YjjJ with different concentration of arabinose. Cells were streaked on LB and LB-chloramphenicol plates and percentage was calculated as survivor on selection plates over plain LB agar plates. **B)** HipB complementation of YjjJ. *hipB* was cloned under the control of an IPTG-inducible promoter while *yjjJ* expression was under an arabinose-inducible promoter. MG1655 strains carrying pEG220 and pBAD33 (Empty vectors), pEG220 and pBAD33::*yjjJ* (Empty vector + YjjJ) or pEG220::*hipB* and pBAD33::*yjjJ* (HipB + YjjJ) were grown on LB to OD_{600} of 0.3 followed by addition of 0.1 mM IPTG and 0.1% arabinose. **C)** Antibiotic tolerance towards ampicillin of MG1655 strains bearing empty vector, pBAD33::*yjjJ* (YjjJ) or pBAD33::*hipA* (HipA). Genes *yjjJ* and hipA are under control of an arabinose-inducible promoter. Strains were grown in LB and plasmid expression was induced at OD_{600} of 0.3, with 0.01% for YjjJ expressing cells and 0.2% arabinose for empty vector and HipA expressing cells for 1 hour, followed by treatment with 100 µg/ml ampicillin. Cells were then harvested at different time points. Growth was followed via optical density and colony forming units. **D)** Micrographs of cells before, one hour and two hours after arabinose induction as in Fig.1. Scale bar: 5 µm.

Fig.S1
