# Supplementary material for: E. coli Toxin YjjJ (HipH) Is a Ser/Thr Protein Kinase That Impacts Cell Division, Carbon Metabolism, and Ribosome Assembly
Source: mSystems. 2022 Dec 20;8(1):e01043-22. doi: 10.1128/msystems.01043-22 (PMC9948734; doi:10.1128/msystems.01043-22)
Supplement: FIG S2 [file msystems.01043-22-s0003.pdf]

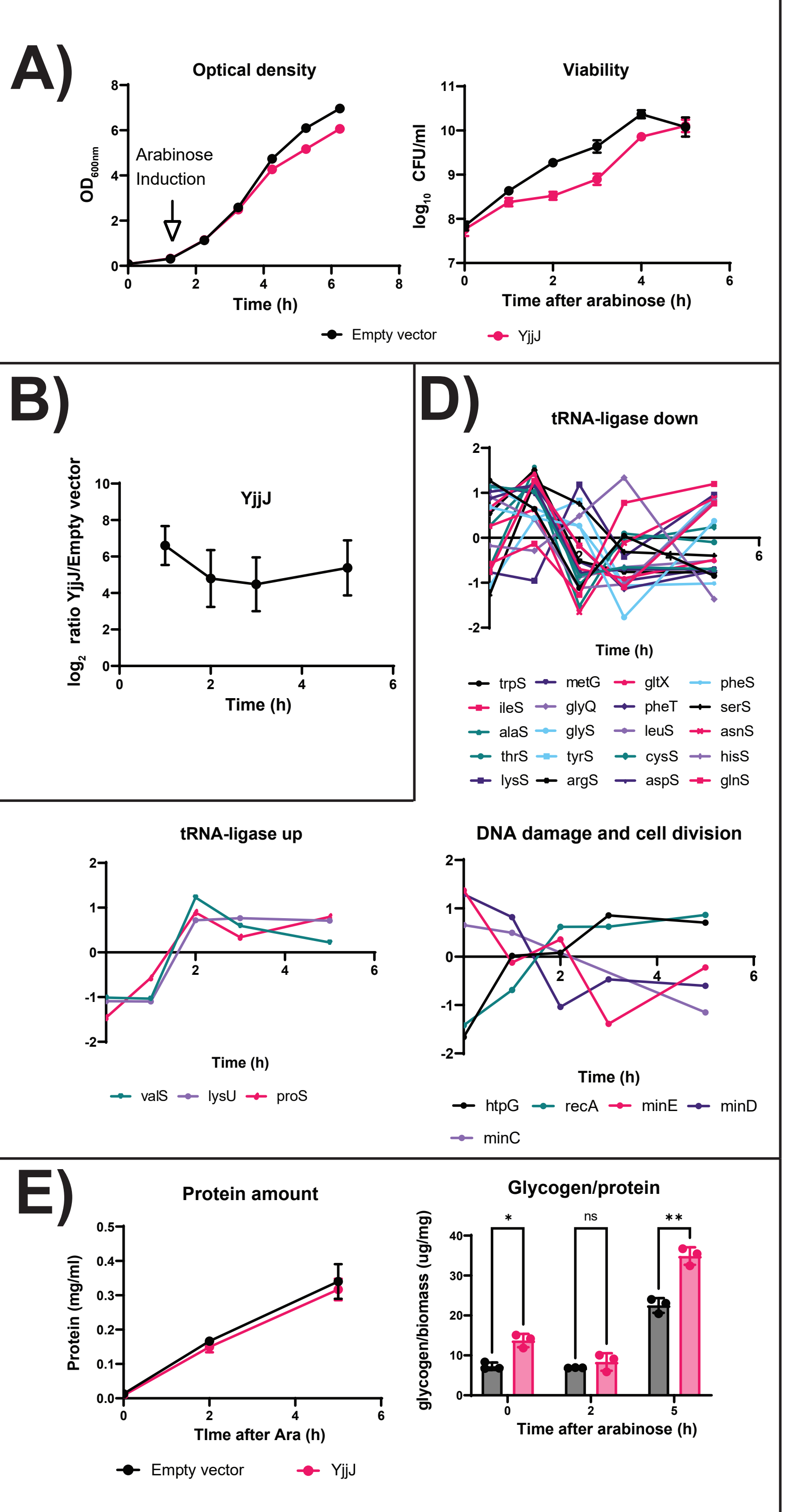

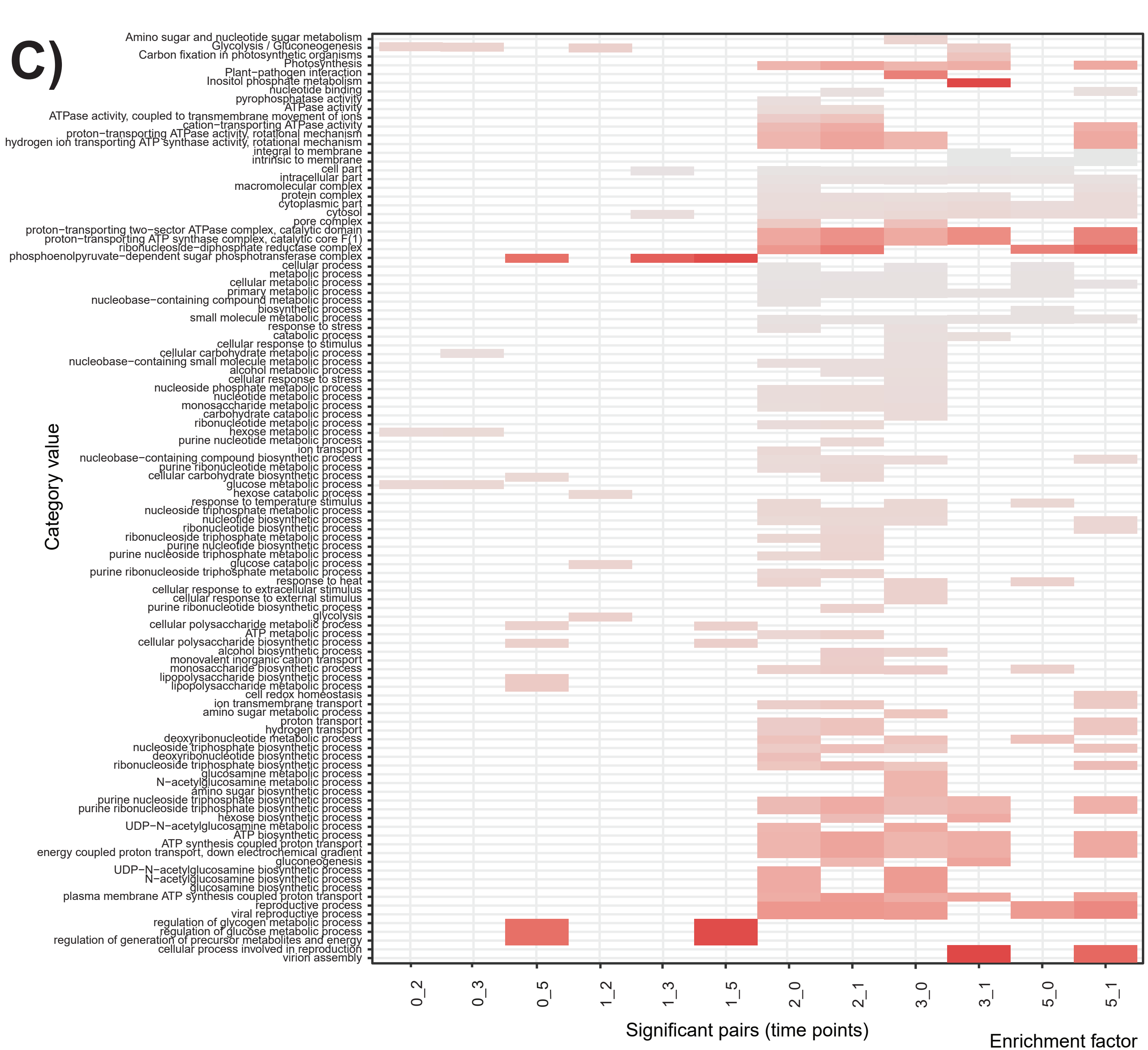

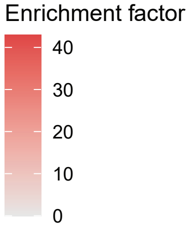

Fig.S2: Prolonged overproduction of YjjJ impacts growth and several physiological pathways.
A) Growth curves of MG1655 strains carrying either empty pBAD33 (Empty vector) or pBAD33::*yjjJ* (YjjJ), in which *yjjJ* expression was under the control of an arabinose-inducible promoter. Strains were grown in LB and expression was induced at OD_{600} of 0.3, with 0.01% arabinose. Growth was followed via optical density and CFU measurements. **B)** Relative abundance of YjjJ during growth, as log_2 ratio between cells bearing empty vector and YjjJ-expressing plasmid. **C)** Gene ontology (GO) enrichment of proteins based on change of abundance among different time points. **D)** Profiles (log_2 ratio YjjJ/Empty vector) over time of selected proteins, as example of proteome dynamics. **E)** Absolute quantification of protein amount and glycogen normalized to the protein content after arabinose induction (0.01%).

Fig.S2
