# Supplementary material for: E. coli Toxin YjjJ (HipH) Is a Ser/Thr Protein Kinase That Impacts Cell Division, Carbon Metabolism, and Ribosome Assembly
Source: mSystems. 2022 Dec 20;8(1):e01043-22. doi: 10.1128/msystems.01043-22 (PMC9948734; doi:10.1128/msystems.01043-22)
Supplement: FIG S3 [file msystems.01043-22-s0004.pdf]

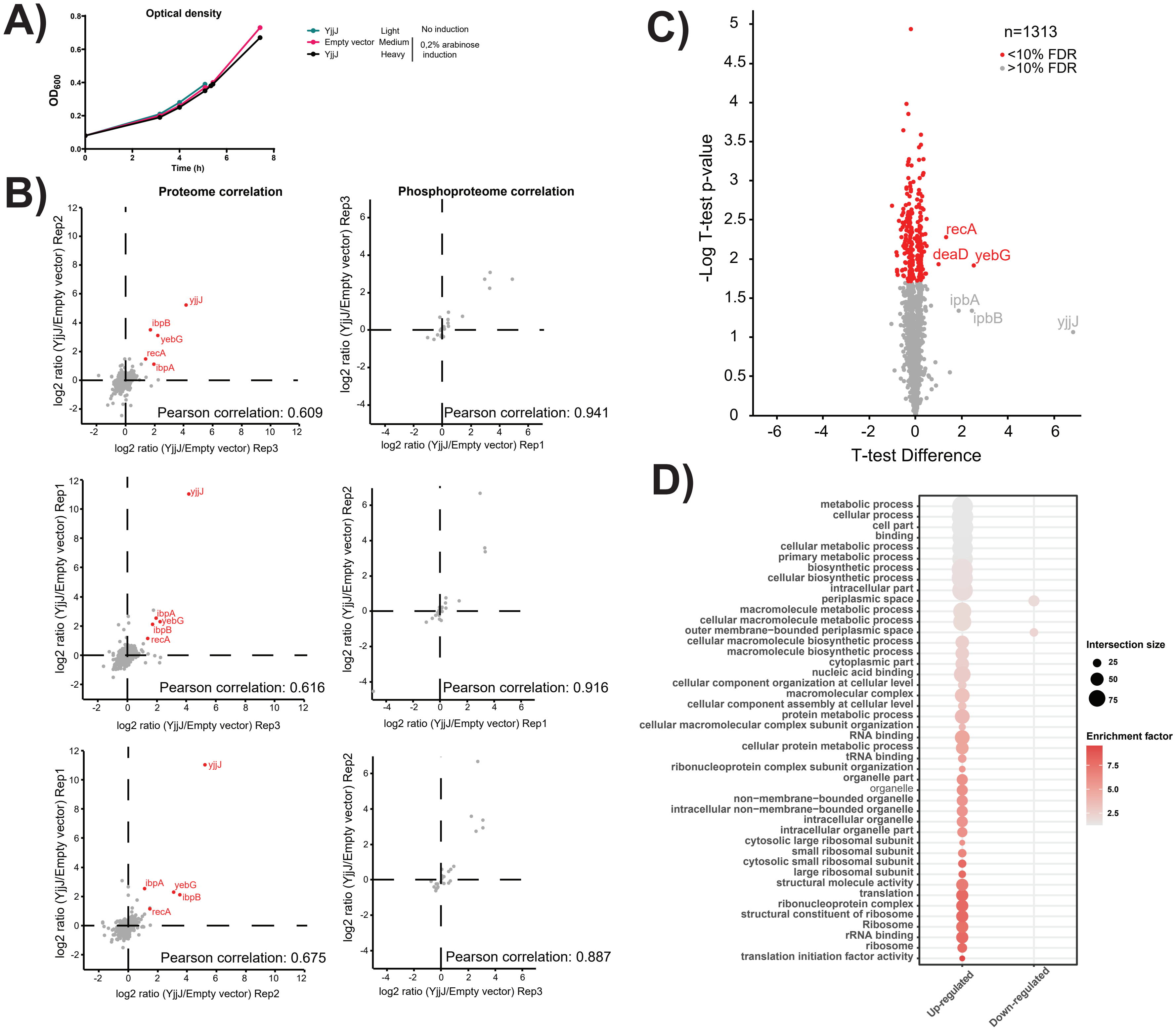

Fig.S3: YjjJ impacts proteome and phosphoproteome with good reproducibility.
A) Growth curves of *E. coli* K-12 MG1655 carrying the pBAD33::*yjjJ* plasmid (YjjJ), in which *yjjJ* expression is under the control of an arabinose-inducible promoter, or pBAD33 as empty vector control. Strains were grown in SILAC-labelled minimal medium containing stable isotope labelled lysine derivatives: "light" lysine (Lys0), "medium-heavy" lysine (Lys4), or "heavy" lysine (Lys8). **B)** Correlation of proteins and phosphorylation-site SILAC ratios from the *yjjJ* expressing strain relative to the strain bearing the empty vector for the three independent replicates. **C)** Volcano plot representing distributions of quantified proteins amongst the three replicates. Proteins with a significant increase (FDR<0.1) are marked in red. **D)** Gene ontology (GO) distribution of those proteins showing an increase in abundance 2 hours after *yjjJ* expression, enriched against the background of all identified proteins.

Fig.S3
