# Supplementary material for: E. coli Toxin YjjJ (HipH) Is a Ser/Thr Protein Kinase That Impacts Cell Division, Carbon Metabolism, and Ribosome Assembly
Source: mSystems. 2022 Dec 20;8(1):e01043-22. doi: 10.1128/msystems.01043-22 (PMC9948734; doi:10.1128/msystems.01043-22)
Supplement: FIG S4 [file msystems.01043-22-s0005.pdf]

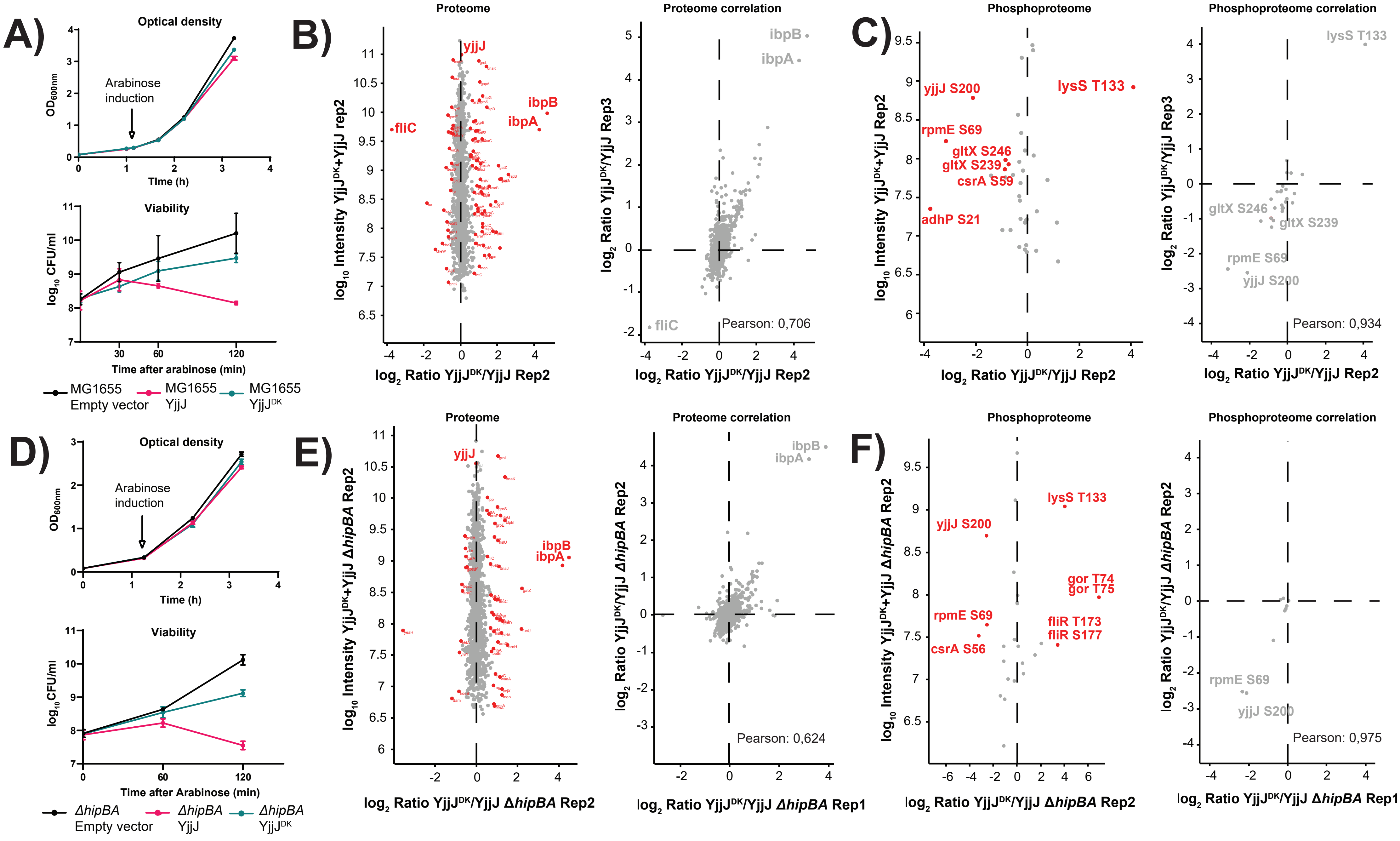

Fig. S4: YjjJ induction cross-talks with HipA pathways, leading to phosphorylation of GltX.
A) Growth curves of *E. coli* K-12 MG1655 wild type carrying the pBAD33::*yjjJ* or pBAD33::*yjjJ S342,364Q* (YjjJ^{DK}) plasmid, in which gene expression is under the control of an arabinose-inducible promoter, or pBAD33 as empty vector control. Strains were grown in LB medium. After reaching OD_{600} of 0.3, plasmid expression was induced with 0.2% arabinose. Growth was followed at OD_{600} and CFU level. **B)** Quantified proteins represented as log_2 ratio between YjjJ native and YjjJ^{DK} expressing cells. Significantly changing proteins ($p<0.05$) are indicated (red) with good correlations between the two replicates. **C)** Distribution of phosphorylation sites upon YjjJ overproduction based on log2 ratio between native YjjJ and YjjJ^{DK} expressing cells shows good correlation between the two replicates. **D)** Growth curve of *E. coli ΔhipBA* carrying either pBAD33::*yjjJ* or pBAD33::*yjjJ S342,364Q* (YjjJ^{DK}) plasmid. Growth was followed at OD_{600} and CFU level. **E)** Quantified proteins represented as log_2 ratio between native YjjJ and YjjJ^{DK} expressing cells in Δ*hipBA* background. Significantly changing proteins ($p<0.05$) are indicated (red) with good correlations between the two replicates. **F)** Distribution of phosphorylated sites upon YjjJ overproduction in the Δ*hipBA* background, based on log_2 ratio between native YjjJ and YjjJ^{DK} expressing cells shows good correlation between the two replicates.

Fig.S4
