# Supplementary material for: E. coli Toxin YjjJ (HipH) Is a Ser/Thr Protein Kinase That Impacts Cell Division, Carbon Metabolism, and Ribosome Assembly
Source: mSystems. 2022 Dec 20;8(1):e01043-22. doi: 10.1128/msystems.01043-22 (PMC9948734; doi:10.1128/msystems.01043-22)
Supplement: FIG S5 [file msystems.01043-22-s0006.pdf]

A)

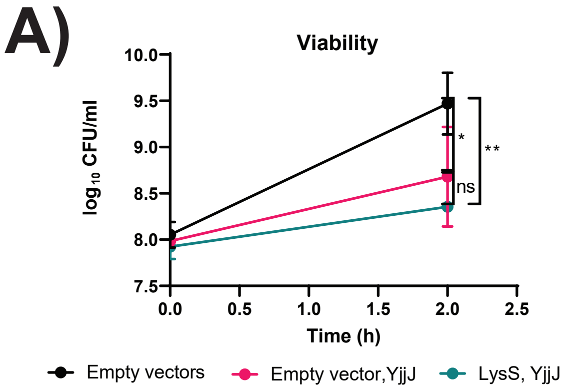

C)

Reaction	RpmE	+	+				
	CsrA			+	+	+	+
	CsrB mRNA					+	+
	ATP		+		+		+
P-site detected	RpmE S69						
	CsrA S56						

B)

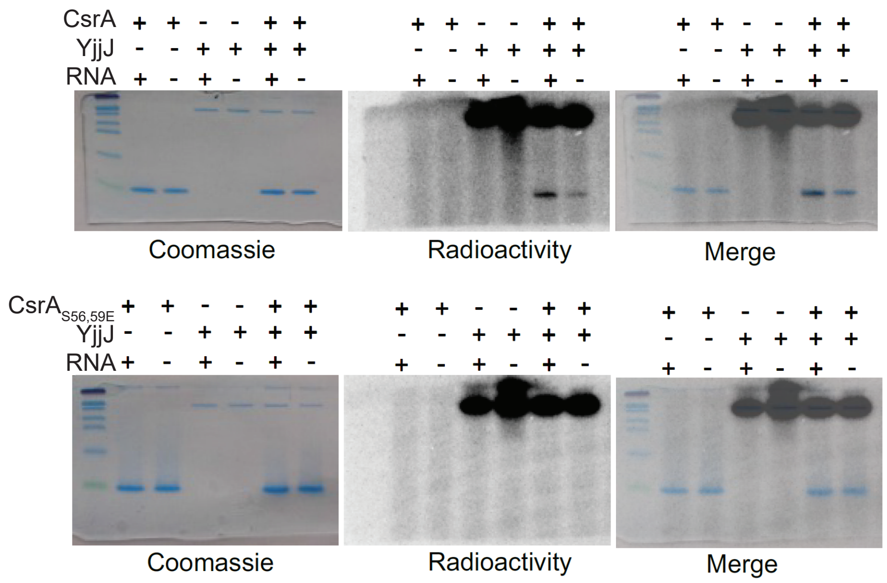

Fig.S5: CsrA phosphorylation is confirmed by *in vitro* kinase autoradiography, indicating mRNA as cofactor.

A) LysS complementation of YjjJ. *lysS* was cloned under the control of an IPTG-inducible promoter while *yjjJ* expression was driven by the arabinose-inducible promoter. MG1655 strains carrying pEG50 and pBAD33 (Empty vectors), pEG220 and pBAD33::*yjjJ* (Empty vector + YjjJ) or pEG25::*lysS* and pBAD33::*yjjJ* (LysS + YjjJ) were grown on LB to OD_{600} of 0.3, followed by addition of 1 mM IPTG and 0.1% arabinose. **B)** *in vitro* phosphorylation of CsrA. Purified YjjJ was incubated with [γ^{32P}] ATP, in the presence or absence of CsrC mRNA. The figure depicts the gel was Coomassie stained, radiograms of the gels and the merge. The negative control is represented by CsrA S56,59E mutant. **C)** Control reactions of *in vitro* kinase assay, measured by mass spectrometry, of Fig.2.

Fig.S5
