# Supplementary material for: E. coli Toxin YjjJ (HipH) Is a Ser/Thr Protein Kinase That Impacts Cell Division, Carbon Metabolism, and Ribosome Assembly
Source: mSystems. 2022 Dec 20;8(1):e01043-22. doi: 10.1128/msystems.01043-22 (PMC9948734; doi:10.1128/msystems.01043-22)
Supplement: FIG S6 [file msystems.01043-22-s0007.pdf]

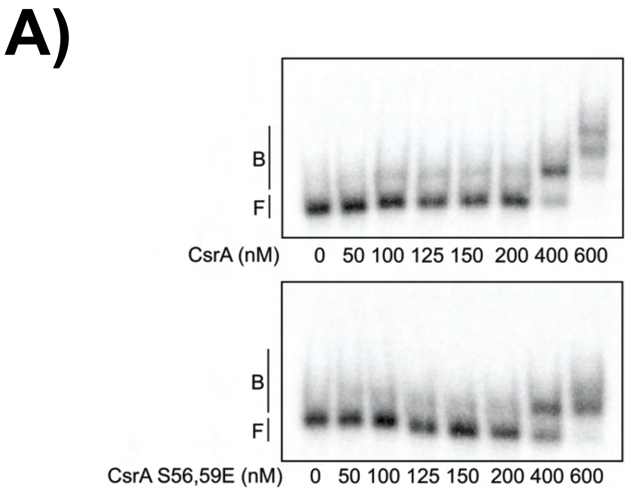

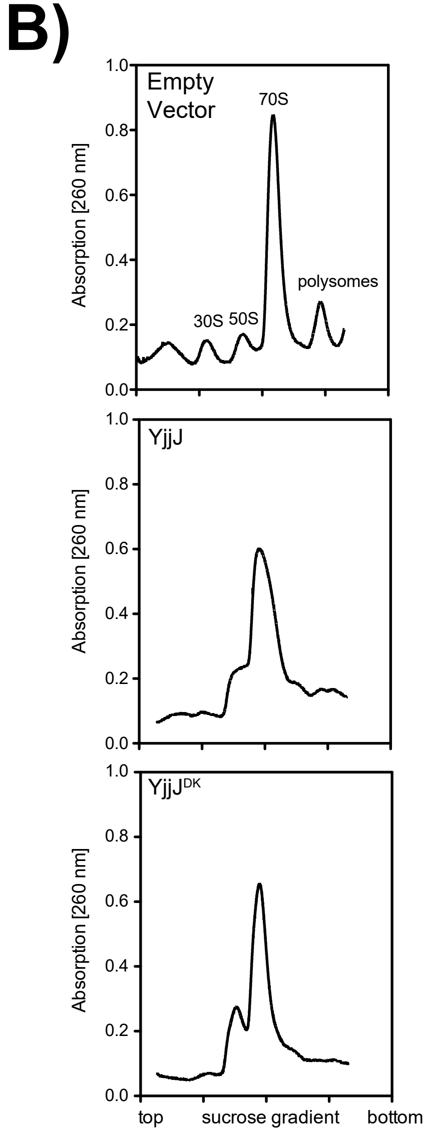

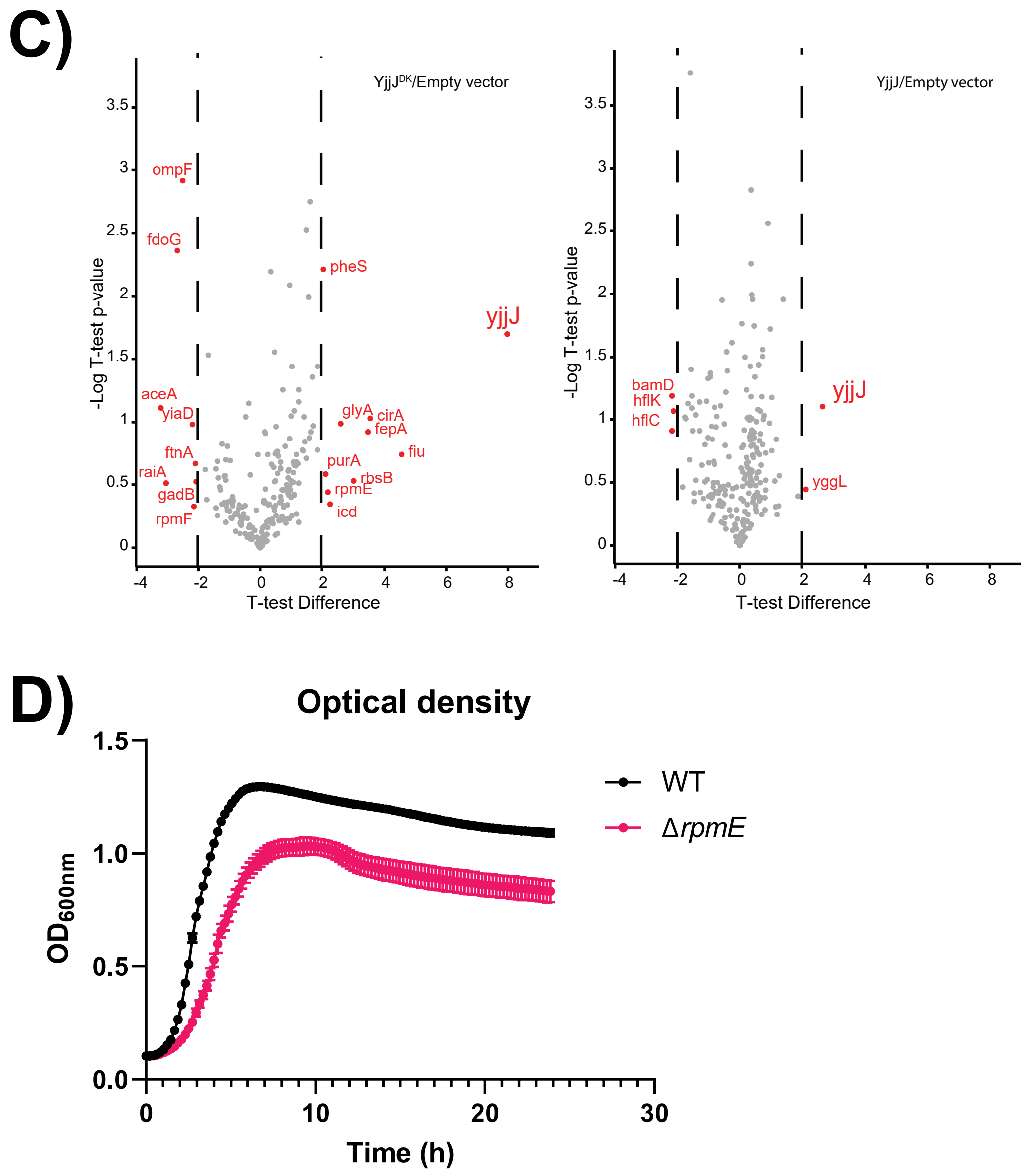

Fig.S6: Control experiments confirm toxicity of YjjJ and its interaction with ribosome.
A) Electromobility assay of CsrA. Different amounts of native and phosphomimetic (S56,59E) CsrA were incubated with CsrC mRNA to test its interaction. F represents unbound and B represents bound mRNA. **B)** Second replicate of ribosome assembly profile from Fig.4C. **C)** MS analysis of crude ribosome extract from ribosome assembly profile experiment. Distributions of proteins represented as log$_2$ ratio between YjjJ^{DK}/Empty vector and YjjJ/Empty. Protein presenting four-fold change of abundance are indicated (red). **D)** Growth comparison between MG1655 wild type and Δ*rpmE* as control for Fig 4C.

Fig.S6
