# Supplementary material for: E. coli Toxin YjjJ (HipH) Is a Ser/Thr Protein Kinase That Impacts Cell Division, Carbon Metabolism, and Ribosome Assembly
Source: mSystems. 2022 Dec 20;8(1):e01043-22. doi: 10.1128/msystems.01043-22 (PMC9948734; doi:10.1128/msystems.01043-22)
Supplement: FIG S7 [file msystems.01043-22-s0008.pdf]

A)

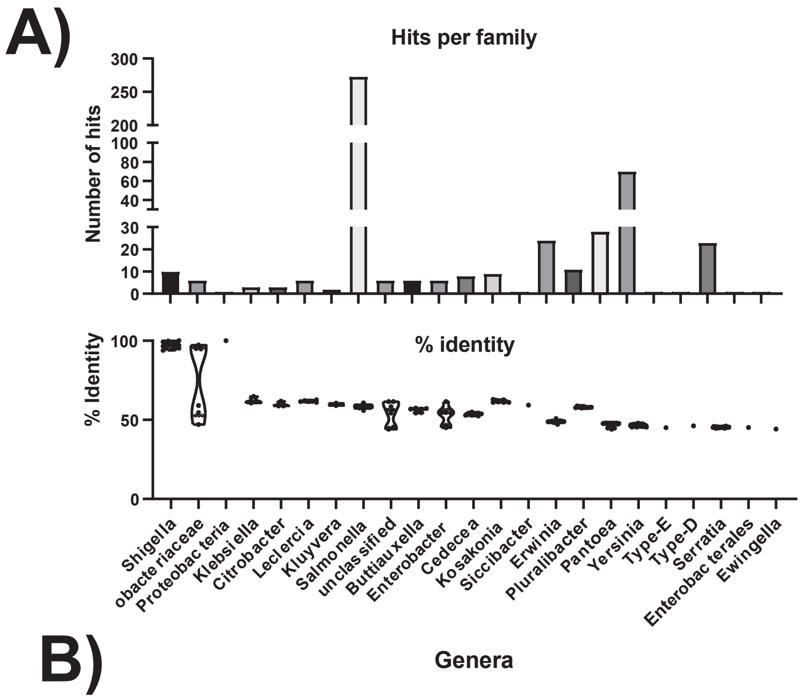

B)

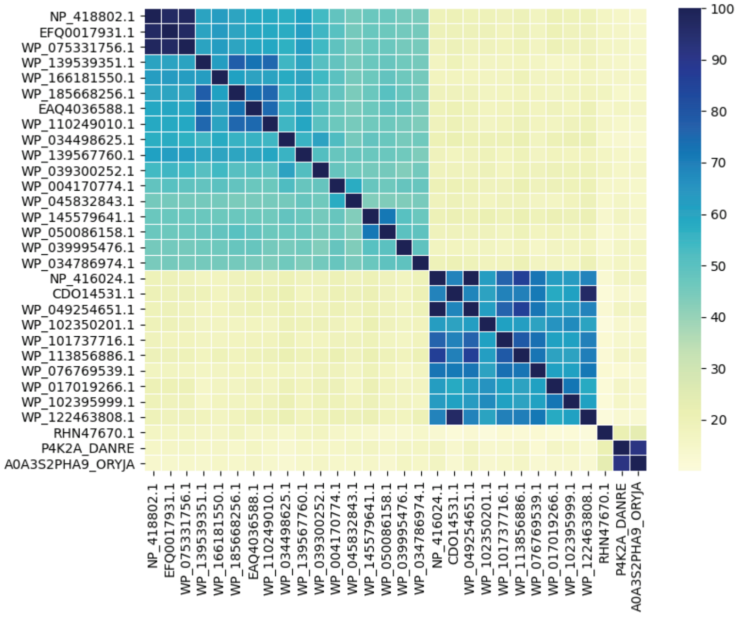

C)

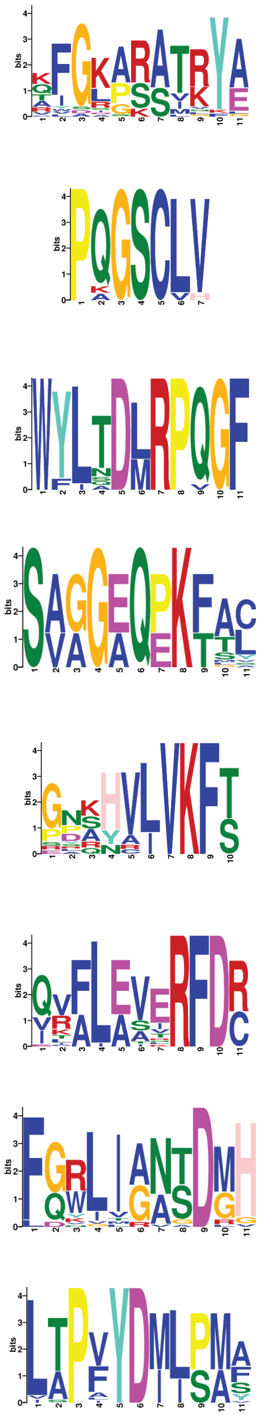

D)

```
YjjJ     1 MSELTDLLLQGPRSAPELRQRLAISQATFSRLVAREDRVIRFGKARATRY     50

HipA     1 --------------------------------------------------      0

YjjJ    51 ALLRPYRGIERIPVW----RVDDTGK----AHKF------------ADIR     80
                   :.::..|    ||.:..|    ||.|            ..:.
HipA     1 --------MPKLVTWMNNQRVGELTKLANGAHTFKYAPEWLASRYARPLS     42

YjjJ    81 LCWPQGSCLVTGADGDEQWFDGLPWYLTD---LRPQGFLGRAWGRKLAAQ    127
           |..|.....:| :|....:||.|   |.|   :|.:  :.:.:..|....
HipA    43 LSLPLQRGNIT-SDAVFNFFDNL---LPDSPIVRDR--IVKRYHAKSRQP     86

YjjJ   128 LNLTDDIRLWQEEDVLYALTVFNGEYTGGWLVGEGNYQRWITAQHP----    173
           .:|..:|    ..|.:.|:|          |:.|..     |..||
HipA    87 FDLLSEI----GRDSVGAVT----------LIPEDE-----TVTHPIMAW    117

YjjJ   174 ---AEIPLDQKLTHYEQLASDALAGEIVG------SSAGGEQ--------    206
              .|..|::.||.|:   :|...|.|..      |.||.::
HipA   118 EKLTEARLEEVLTAYK---ADIPLGMIREENDFRISVAGAQEKTALLRIG    164

YjjJ   207 -----PK----FTYYAQTPSGNKHVLVKFTVPQQT-----AVSQRWGDLL    242
                ||    .|:..:.|.|      :...|..|     :|...:..||
HipA   165 NDWCIPKGITPTTHIIKLPIG------EIRQPNATLDLSQSVDNEYYCLL    208

YjjJ   243 IAESIA-----AQILRDGGIHAIESTVLVTSNRQVFLEAERFDCKGNDGR    287
           :|:.:.     |:|::.|.:.|              |..||||.:.|..|
HipA   209 LAKELGLNVPDAEIIKAGNVRA--------------LAVERFDRRWNAER    244

YjjJ   288 LPIVSL------------EAVQSEFISSPGSWPQAMRRL------CEQQL    319
           ..::.|            .:|:.|....||     :.|:      ..:.|
HipA   245 TVLLRLPQEDMCQTFGLPSSVKYESDGGPG-----IARIMAFLMGSSEAL    289

YjjJ   320 VTHQSVAQTEVIWAFGRLIANSDMHAGNLSFYL-SEPPFALTPVYDMLPM    368
           .......:.:|   |..||..:|.||.|.|.:: :...:.|||.||::..
HipA   290 KDRYDFMKFQV---FQWLIGATDGHAKNFSVFIQAGGSYRLTPFYDIISA    336

YjjJ   369 VYAPNSAGMLRDAAIEVKFDLNVSKSAWLTAIPLAQQFWQTVARDPRISE    418
           .......| :..:.::...||.||.........:..:.:...|:..|..|
HipA   337 FPVLGGTG-IHISDLKLAMGLNASKGKKTAIDKIYPRHFLATAKVLRFPE    385

YjjJ   419 AFRH--------------------IAQEMPEK-IRQIEEKVARMGG----    443
           ...|                    :..:.||. :..:|..|.|:.|
HipA   386 VQMHEILSDFARMIPAALDNVKTSLPTDFPENVVTAVESNVLRLHGRLSR    435

YjjJ   444 -----    443

HipA   436 EYGSK    440
```

Fig.S7: A) Distribution of *yjjJ* among different genera (upper panel) and percentage of identity (lower panel). **B)** Multiple sequence alignment (Clustal Omega) of 16 different YjjJ, 13 HipA and three ¾ kinase protein sequences from various organisms. **C)** Visualization of motifs identified in multiple sequence alignment. **D)** Pairwise sequence alignment between YjjJ and HipA proteins.

Fig.S7
