# Supplementary material for: E. coli Toxin YjjJ (HipH) Is a Ser/Thr Protein Kinase That Impacts Cell Division, Carbon Metabolism, and Ribosome Assembly
Source: mSystems. 2022 Dec 20;8(1):e01043-22. doi: 10.1128/msystems.01043-22 (PMC9948734; doi:10.1128/msystems.01043-22)
Supplement: FIG S8 [file msystems.01043-22-s0009.pdf]

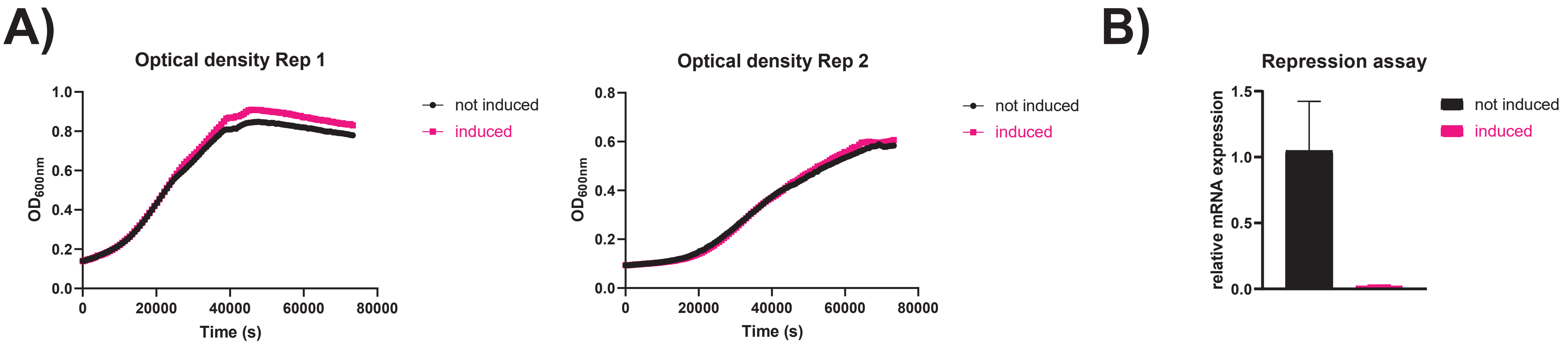

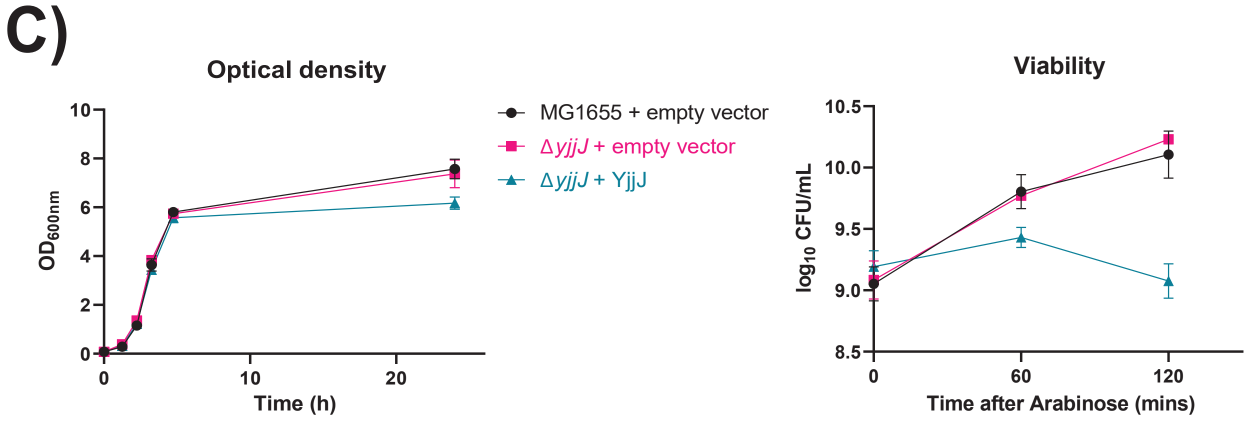

D)

Gene name	Intensity L (MG1655 + pBAD33)	Intensity M (Δ*yjjJ* + pBAD33)	Intensity H (Δ*yjjJ* + YjjJ)
yjjJ Rep1	3.94E+08	0	4.01E+10
yjjJ Rep2	4.83E+08	0	2.25E+10

Fig.S8: Effect of CRISPRi-Silencing and Deletion of YjjJ on growth.
A) Growth curve of *E. coli* strain *YYdCas9:BW25993* cells with *yjjJ*-pgRNA. Expression of dCas9 induced by aTc 100ng/ml to repress *yjjJ* or not induced (as a control). Cells were grown in M9 minimal medium in 24-well microtiter plates. Growth was followed in two independent experiments. Samples harvested at the end of the growth curve for repression assay. **B)** Repression assay for relative mRNA levels of YjjJ induced vs not induced by doing real-time PCR with cDNA. Data is normalized to the expression of the endogenous control gene, *hcaT*. **C)** Growth curve of *E. coli K-12* MG1655 carrying the empty vector and *E. coli ΔyjjJ* carrying either the empty vector or pBAD33::*yjjJ* plasmid (YjjJ) in which *yjjJ* expression was under the control of an arabinose-inducible promoter. Strains were grown in LB medium and expression was induced at OD_{600} of 0.3, with 0.2% arabinose. Growth was followed via optical density and CFU measurements. **D)** Table for confirmation of knockout via MS measurements. Strains were grown in SILAC-labelled minimal medium containing stable isotope labelled lysine derivatives: “light” lysine (Lys0), “medium-heavy” lysine (Lys4), or “heavy” lysine (Lys8), harvested after 2 hrs of induction with 0.2% arabinose , followed by sample preparation and measurement via MS.

Fig.S8
