# Supplementary material for: E. coli Toxin YjjJ (HipH) Is a Ser/Thr Protein Kinase That Impacts Cell Division, Carbon Metabolism, and Ribosome Assembly
Source: mSystems. 2022 Dec 20;8(1):e01043-22. doi: 10.1128/msystems.01043-22 (PMC9948734; doi:10.1128/msystems.01043-22)
Supplement: TEXT S1 [file msystems.01043-22-s0001.pdf]

SUPPLEMENTARY MATERIAL AND METHODS

Bacterial strains and plasmids

Strains, plasmids and primers used in this work are listed in table. Due to high homology with HipA, *yjjJ* gene was cloned with same Shine-Dalgarno sequence used in previous work (1). Genes were PCR-amplified from genomic DNA of *E. coli*. Clonings were performed via Gibson assembly (2), any exception is explained separately . For insertion of PCR-amplified gene fragment: pET28a vector was digested with NdeI, pBAD33 vector XbaI and pEG25 with BamHI. Point mutations of *yjjJ* and *rpmE* were performed using QuikChange II Site-Directed Mutagenesis kit (Agilent, 200523). *E. coli* gene coding for CsrA was PCR-amplified from genomic DNA of *E. coli* K12. XhoI and NcoI restriction sites were introduced during amplification using appropriate primers (Extended Data Table 3). The resulting PCR product was digested with XhoI and NcoI and cloned into pET-28c vector (Merck Millipore). The CsrA S56,59E mutants was generated by site-directed mutagenesis using a procedure based on the Phusion Site-Directed Mutagenesis Kit (Thermo Scientific). The resulting plasmids were sequenced and transformed in the final working strains.

Primer list

SD8-YjjJ. pBAD33-For	TCGGTACCCGGGGATCCTAAAATAAGGAGGAAAAAAAAAATGAGCGAGCTGACTGATCT	*yjjJ* cloning in pBAD33 vector with Shine Dalgarno sequence in red
YjjJ.pBAD33-rev	CATGCCTGCAGGTCGACTTTACCCGCCCATGCGG	*yjjJ* cloning in pBAD33 vector
SDM_primer_D342->Q_1	ATAAATTACCTGCGTGCATCTGGCTGTTGGCGATAAGTCGC	Site direct mutagenesis for *yjjJ*
SDM_primer_D342->Q_2	GCGACTTATCGCCAACAGCCAGATGCACGCAGGTAATTTAT	Site direct mutagenesis for *yjjJ*
SDM_primer_D364->Q_1	CCATCGGCAGCATCTGGTAGACGGGCGTC	Site direct mutagenesis for *yjjJ*

SDM_primer_D364->Q_2	GACGCCCGTCTACCAGATGCTGCCGATGG	Site direct mutagenesis for *yjjJ*
csrA.pBAD-for	CTCGGTACCCGGGGATCCTAAAATAAGGAGGAAAAAAAAAATGCTGATTCTGACTCGTCG	*csrA* cloning in pBAD33 vector with Shine Dalgarno sequence in red
csrA.pBAD-rev	TGCATGCCTGCAGGTCGACTTTAGTAACTGGACTGCTGGGA	*csrA* cloning in pBAD33 vector with
Rev SDM_CsrA_Serine 56 59	ATACGCTGGTAGATCTCTTCACGGTGAACAGA	Site direct mutagenesis for *csrA*
Fwd SDM_CsrA_Serine S56E	CCAGGCTGAAAAAGAACA GCAGTCCAGTTACGGTG	Site direct mutagenesis for *csrA*
Fwd SDM_CsrA_Serine S59E	CCAGGCTGAAAAATCCCA GCAGGAAAGTTACGGTG	Site direct mutagenesis for *csrA*
Fwd SDM_CsrA_Serine S56E S59E	CCAGGCTG AAAAAGAACA GCAGGAAAGTTACGGTG	Site direct mutagenesis for *csrA*
rpmE_Sdopt_pEG25-for	AGAAATTAACCATGGGAGAAAATAAGGAGGAAAAAAAAATGAAAAAAGATATTCACCCGA	*rpmE* cloning in pBAD33 vector with Shine Dalgarno sequence
rpmE_pEG25-rev	CTTGGCTGCAGGTCGACGTTATTTGCTGCCCGGGATGT	*rpmE* cloning in pBAD33 vector with
rpmE.S69E.pFG11-for	CTG CAG GTC GAC GTT ATT TCT CGC CCG GGA TGT TGA AAC GC	Site direct mutagenesis for RpmE
rpmES69E.pFG11-rev	GCG TTT CAA CAT CCC GGG CGA GAA ATA ACG TCG ACC TGC AG	Site direct mutagenesis for RpmE
YjjJ.pET24a-f	CACCAGTCATGCTAGCCATTACCCGCCCATGCGG	*yjjJ* cloning in pET24a vector
YjjJ.pET24a-r	GGTGCCGCGCGGCAGCCATATGAGCGAGCTGACTGATCT	*yjjJ* cloning in pET24a vector
rpmE.pET24a-f	GGTGCCGCGCGGCAGCCAATGAAAAAAGATATTCACCCGA	*csrA* cloning in pET24a vector
rpmE.pET24a-r	CACCAGTCATGCTAGCCATTATTTGCTGCCCGGGATGT	*csrA* cloning in pET24a vector
Fwd_CsrA_NcoI	ATCGACCCATGGGACTGATTCTGACTCGTCGAGTTGGTGAG	*csrA* cloning in pET28a vector
Rev_Csra_Tev_XhoI	GTGCTCGAGGCCCTGAAAATAAAGATTCTCACCGTAACTGGACTGCTGGGATTTTTCAGC	*csrA* cloning in pET28a vector
YjjJ.seq-for	GTCTCTGGCAGGAAGAAGATG	Sequencing primer for *yjjJ*
YjjJ.seq-rev	ACAAGCACCGTTGACTCG	Sequencing primer for *yjjJ*
pET28a.seq-f	GGGGAATTGTGAGCGGAT	Sequencing primer for pET28a
pet28a.seq-r	CAGCCAACTCAGCTTCCT	Sequencing primer for pET28a

pBAD F	CGCAACTCTCTACTGTTTCTC	Sequencing primer for pBAD33
pBAD R	CCGCTTCTGCGTTCTG	Sequencing primer for pBAD33
pEG25_seq-for	GCCCTTTCCTGTTCAGCTC	Sequencing primer for pEG25
pEG25_seq-rev	GTAGCGCCGGAAGATGCTTT	Sequencing primer for pEG25
yjjJ_Ec-F-fwd	CGCAATTCCGGGGCAGAACGGTTTTAGAGCTAGAAATAGCAAGTTAAAATAAGGC	Primer for yjjJ sg-RNA (in red) for amplification of pgRNA
Ec-F-rev	ACTAGTATTATACCTAGGACTGAGCTAGC	Primer for amplification of pgRNA
hcaT-FWD	CGC TCG GCT ATT TCA CAT ACT	RT-PCR primer for hcaT cDNA
hcaT-REV	GGT TTC TGG CGT TAA ACC AAT C	RT-PCR primer for hcaT cDNA
yjjJ-FWD	ACC GGA AAG GCG CAT AAA	RT-PCR primer for yjjJ cDNA
yjjJ-REV	GGT CGG AGA TCG GTC AAA TAC	RT-PCR primer for yjjJ cDNA

Strain list

MG1655	F- λ- ilvG- rfb-50 rph-1	*E. coli* Genetic StockCenter, CGSC no. 7740
BL21 (DE3)	*fhuA2 [lon] ompT gal (λ DE3) [dcm] ΔhsdS λ DE3 = λ sBamHIo ΔEcoRI-B int::(lacI::PlacUV5::T7 gene1) i21 Δnin5*	New England Biolabs
JW3907-1 (KEIO strain)	*F-, Δ(araD-araB)567, ΔlacZ4787(::rrnB-3), λ-, rph-1, Δ(rhaD-rhaB)568, ΔrpmE723::kan, hsdR514*	(3)
JW4348-1 (KEIO strain)	*F-, Δ(araD-araB)567, ΔlacZ4787(::rrnB-3), λ-, rph-1, Δ(rhaD-rhaB)568, hsdR514, ΔyjjJ781::kan*	(3)
MG1655 *ΔyjjJ*	MG1655 *ΔyjjJ781::kan*	This work
MG1655 *ΔrpmE*	MG1655 *ΔrpmE723::kan*	This work
MG1655 *ΔhipBA*	*hipBA::FRT*	(4)
Top10	*mcrA, Δ(mrr-hsdRMS-mcrBC), Phi80lacZ(del)M15, ΔlacX74, deoR, recA1, araD139, Δ(ara-leu)7697, galU, galK, rpsL(SmR), endA1, nupG*	Invitrogen

XL10-Gold Ultracompetent Cells	*Tetr Δ(mcrA)183 Δ(mcrCB-hsdSMR-mrr)173 endA1 supE44 thi-1 recA1 gyrA96 relA1 lac Hte [F´ proAB lacIqZΔM15 Tn10 (Tetr) Amy Camr]**	Agilent
YYdCas9:BW25993 with CRISPRi	*intC::TetR-dcas9-aadA laqY::ypet-cat*	(5)
YYdCas9:BW25993 with CRISPRi-pgRNA-*yjjJ*	*intC::TetR-dcas9-aadA laqY::ypet-cat*	This work

Plasmid list

pBAD33	*p15, cat, araC,* P_{BAD} *promoter, Cm*	(6)
pBAD33::*yjjJ*	pBAD33 P_{BAD}:: *sd8 gtg yjjJ*	This work
pBAD33::*hipA*	pBAD33 P_{BAD}:: *sd8 gtg hipA*	(4)
pBAD33::$yjjJ^{DK}$	pBAD33 P_{BAD}:: *sd8 gtg yjjJ D342Q D364Q*	This work
pBAD33::*csrA*	pBAD33 P_{BAD}:: *sd8 csrA*	This work
pBAD33::*csrA S56E*	pBAD33 P_{BAD}:: *sd8 csrA S56E*	This work
pEG25	*pUC, bla,* P_{lac} *promoter, AmpR*	Gerdes lab
pEG25::*rpmE*	pEG25 P_{lac}:: *rpmE*	This work
pEG25::*rpmE S69E*	pBAD33 P_{lac}:: *rpmE S69EJ*	This work
pET28a	T7lac promoter, His tag, T7 tag, KmR	New England Biolabs
pET28a::*yjjJ*	pET28a:: *6his yjjJ*	This work
pET28a::$yjjJ^{DK}$	pET28a:: *6his yjjJ D342Q D364Q*	This work
pET28a::*rpmE*	pET28a:: *6his rpmE*	This work
pET28a::*csrA*	pET28a:: *6his csrA*	This work
pET28a::*csrA S56E*	pET28a:: *6his csrA S56E*	This work
pET28a::*csrA S59E*	pET28a:: *6his csrA S59E*	This work
pET28a::*csrA S56E S59E*	pET28a:: *6his csrA S56E S59E*	This work
pNDM220	Mini-R1, bla, lacIq , PA1/O4/O3, Amp	(7)
pNDM220::*hipB*	pNDM220 P_{lac}*::sdopt::hipB*	(1)
pgRNA-cntrl	pgRNA_cntrl	(8)
yjjJ-pgRNA	pgRNA_yjjJ	This work

Bioinformatic analysis

A psiBLAST was performed at the NCBI server (https://blast.ncbi.nlm.nih.gov/Blast.cgi). In this search the *E. coli* organism was fully excluded in all iterations. One subsequent iteration of psiBLAST with the top 500 hits was performed. Identity and number of hits per family were extracted from the BLAST results and graphs generated with PRISM software. From the

second psiBLAST iteration, and YjjJ protein relatives, 16 YjjJ, 13 HipA and three ¾ kinase proteins from various organisms were selected for subsequent analysis for a total of 30 proteins. The proteins were aligned using the EBI implementation of Clustal Omega with default settings (https://www.ebi.ac.uk/Tools/msa/clustalo/). The pairwise similarity of all sequences against each other were printed to a heatmap using the seaborn python library. To find motifs in the MSA, the MEME software was used (http://meme-suite.org) with a 4th order markov model searching for 8 motifs in the length range of 6 to 20 amino acids which were represented as logos. Pairwise sequence alignment, between YjjJ and HipA, proteins was performed with EMBOSS Needle (https://www.ebi.ac.uk/Tools/psa/emboss_needle/), using the software pre-settings.

Growth experiments

Pre-cultures were grown overnight in liquid medium (Luria-Broth or M9 minimal medium) supplemented with 0.4% (w/v) glucose, 25 µg/ml chloramphenicol, for maintenance of pBAD33 plasmids, 25 µg/ml kanamycin, for pET28 plasmids, and 100 µg/ml ampicillin, for pEG25 vectors. Day culture were inoculated at 0.08 OD_{600} and once reached 0.3 OD_{600}, arabinose was added at the desired concentration. Antibiotic tolerance experiments were performed with the same strategy, adding 0.01% arabinose once culture reach OD_{600} of 0.3, for one hour, followed by antibiotic addition (100 µg/ml for ampicillin and 1 µg/ml for ciprofloxacin). Sample were harvest at the indicated time points, pellets were washed with phosphate-saline buffer (PBS), serially diluted and spotted on LB-agar plates supplemented with 0.4% (w/v) glucose. Experiments were performed in triplicates and results visualized using Prism8 (GraphPad). Plasmid loss analysis were performed by streaking out a minimum

of 12 single colonies from the triplicates of growth experiment, on LB and LB supplemented with chloramphenicol plates. Percentage was calculated as number of colonies surviving on selective plates over LB plain agar plates.

SILAC labeling

For quantitative phosphoproteomic experiments, *E. coli* cells were differentially labeled using stable isotope–labeled lysine derivatives: 4,4,5,5-D4 l-lysine (Lys4, medium-heavy lysine, K4, Cambridge Isotope Laboratories), 13C6 15N2 l-lysine (Lys8, heavy lysine, K8, Cambridge Isotope Laboratories) and l-lysine (Lys0, light lysine, K0, Sigma-Aldrich) (9). Precultures and main cultures were grown in M9 minimal medium containing 0.025% (w/v) Lys0, Lys4, or Lys8, precultures were supplemented with 0.4% glucose.

Dimethyl-labelling

In brief, 1ml of the respective labeling solutions with CH_2O (Sigma-Aldrich) and $NaBH_3CN$ (Fluka) for light- and CD_2O (Sigma-Aldrich) and NaBH3CN for medium- labeling were flushed with 2 – 5 min contact time through the stage tip. Labeled peptides were washed with 200 µl HPLC Solvent A (0.5% acetic acid) on the tip and eluted with HPLC Solvent B (80% acetonitrile in 0.5% acetic acid). For validation of labeling efficiency and correct mixing of the labeled peptides, two times 5 µg of each labeled sample (based on Bradford measurements) were used for separate measurements (label efficiency) or mixed 1:1 to pilot LC-MS/MS measurements. Based on the obtained label ratios, correction factors were applied for correct mixing of samples. The labeling efficiencies were in all cases ≥96%.

Cell lysis and protein extraction

Cultures were harvested at specific time points by centrifugation at 4°C and stored at −80°C. The cell pellets were resuspended in a lysis buffer [SDS (40 mg/ml), 100 mM tris-HCl (pH 8.6), 10 mM EDTA, 5 mM glycerol-2-phosphate, 5 mM sodium fluoride, 1 mM sodium orthovanadate, and complete protease inhibitors (Roche) and sonicated till clearness (at least five times for 30 s at 40% amplitude). The cellular debris was pelleted by centrifugation at 13,000g for 30 min, and the crude protein extract was precipitated from the supernatant with methanol and chloroform. Protein pellet was resuspended in a denaturation buffer containing 6 M urea, 2 M thiourea, and 10 mM tris (pH 8.0). Protein concentration was measured using standard Bradford assay (Bio-Rad).

Protein digestion in solution

In each SILAC experiment, differently labeled protein extracts were mixed in equal amounts corrected by the ratios determined by measuring mixing checks (see below) to a total of 12 mg. Proteins were reduced using 1 mM dithiothreitol (DTT) for 1 hour and subsequently alkylated with 5.5 mM iodoacetamide for 1 hour. One-half of the protein mixture was diluted with four volumes of 62.5 mM tris (pH 8.0) and 12.5 mM $CaCl_2$ and digested with chymotrypsin (1:120, w/w) overnight at room temperature (RT). The other half was predigested with endoproteinase Lys-C (1:100, w/w) for 3 hours, then diluted with four volumes of 62.5 mM tris (pH 8.0), and supplemented with endoproteinase Lys-C (1:100, w/w) for overnight digestion at RT. The reaction was stopped by acidification with trifluoroacetic acid (TFA) to pH 2. An aliquot of 10 µg was purified by StageTips (see below), and 2 µg was

used for direct proteome measurement with 230-min LC gradient. An additional aliquot of at least 100 µg intended for further proteome measurements was stored at −80°C. For dimethyl-labelling experiment, samples were digested with trypsin, predigested with endoproteinase Lys-C.

Phosphopeptide enrichment

Digested peptides were desalted by the solid-phase extraction using Sep-Pak C18 Vac 100 mg column (Waters). Briefly, column was activated with methanol and equilibrated with solvent A* [2% (v/v) acetonitrile and 1% (v/v) formic acid]. After loading the sample, the column was washed with solvent A [0.1% (v/v) formic acid], and peptides were eluted with 1.8 ml of 80% (v/v) acetonitrile and 6% (v/v) TFA. Eluted peptides were eluted with 1.8 ml 80% (v/v) acetonitrile and 6% (v/v) TFA. Phosphopeptides were enriched using MagReSyn® Ti^{4+}-IMAC beads with a ratio 1:2.5 peptide to beads ratio, following the manufacture instructions. Beads were washed twice with ethanol 70%, followed by an additional wash step with elution buffer (1% NH_4OH). Beads were then equilibrated three times with loading buffer (1M glycolic acid in 80% acetonitrile and 5% trifluoracetic acid). Samples were incubated with equilibrated beads for 20 minutes at constant shake at room temperature. Supernatant was discarded and the unbound sample was washed away with loading buffer. Three washing steps with wash buffer I (80% acetonitrile, 1% trifluoracetic acid) removed non-specific bound peptides. One additional washing step was performed with an aqueous solution of 10% acetonitrile and 0.2% trifluoroacetic acid. Three rounds of elution of bound peptides was performed using incubating the beads with elution buffer for 15 minutes.

Incorporation and mixing check

For incorporation and mixing check, 10 µg of each sample was separately digested with endoproteinase Lys-C (for SILAC experiment) or trypsin (for dimethyl experiment), purified by StageTips (see below), and measured by LC-MS/MS. In all cases, the labeling efficiencies of all the different labels were ≥94%. Before the mixing of labeled samples for SILAC experiments, 20 µg of each differentially labeled sample was premixed in equal protein amounts determined by Bradford assay, digested with endoproteinase Lys-C, and measured by LC-MS/MS. Median of evidence labelling ratios was used as a correction factor for mixing the samples to be used in SILAC and dimethyl-labelled experiments.

Peptide purification by StageTips

Before each LC-MS/MS measurement, all peptide samples were desalted and purified on C18 StageTips (10). Reversed-phase C18 discs (Empore) were activated with methanol and equilibrated with solvent A*. Up to 10 µg of peptides was loaded onto the membrane and washed with solvent A. Peptides were eluted with 50 µl of solvent B [80% (v/v) acetonitrile and 0.1% (v/v) formic acid] and concentrated by vacuum centrifugation. The sample volume was adjusted with solvent A and final 10% (v/v) of solvent A*.

LC-MS/MS measurement

Purified peptide samples were separated by an EASY-nLC 1000 or 1200 system (Thermo Fisher Scientific) coupled online to a Q Exactive HF mass spectrometer (Thermo Fisher Scientific) through a nanoelectrospray ion source (Thermo Fisher Scientific). Chromatographic

separation was performed on a 20-cm-long, 75–µm–inner diameter analytical column packed in-house with reversed-phase ReproSilPur C18-AQ 1.9 µm particles (Dr. Maisch GmbH). The column temperature was maintained at 40°C using an integrated column oven. Peptides were loaded onto the column at a flow rate of 700 nl/min or 1 µl/min under maximum back pressure of 850 bar. The peptides were eluted using either 60 (proteome dynamics measurement, 0.5 µg) or 230 min (SILAC-based proteome analysis, 2 µg) segmented gradient of 10 to 50% solvent B at a constant flow rate of 200 nl/min. When measuring proteome digested with chymotrypsin, the gradient started with 5% of solvent B. For measurements of kinase assays 0.2 µg material was injected and the peptides were eluted using 44 min segmented gradient of 5 to 50 solvent B at a constant flow rate of 300 nl/min. Peptides were ionized by nanoelectrospray ionization at 2.3 kV and the capillary temperature of 275°C. The mass spectrometer was operated in a data-dependent mode, switching automatically between one full scan and subsequent MS/MS scans of either 12 (Top12 method, for proteome measurements) or 7 (Top7 method, phosphoproteome and *in vitro* kinase measurements) most abundant peaks selected with an isolation window of 1.4 m/z (mass/ charge ratio). Full-scan MS spectra were acquired in a mass range from 300 to 1650 m/z at a target value of 3×10^6 charges with the maximum injection time of 25 ms, 45 ms for *in vitro* kinase assay, and a resolution of 60,000 (defined at m/z 200). To prevent repeated fragmentation, the masses of sequenced precursors were dynamically excluded for 30 s, reduced to 20 s for *in vitro* kinase assay. The higher-energy collisional dissociation MS/MS spectra were recorded with the maximum injection time of 45 or 220 ms (for phosphoproteome and *in vitro* kinase measurements) at a target value of 1×10^5 and a resolution of 30,000 (defined at m/z 200), or 60,000 for phosphoproteome and *in vitro* kinase measurements. The normalized collision energy was set to 27%, and the intensity threshold

was kept at 1×10^5 for proteome, 5×10^4 for phosphoproteome measurement or 4.5×10^4. The masses of sequenced precursor ions were dynamically excluded from MS/MS fragmentation for 30 s. Ions with single, unassigned, or six and higher charge states were excluded from fragmentation selection.

MS data processing and analysis

For all SILAC based experiment we performed same pipeline. Acquired raw data were processed using the MaxQuant software suite (version 1.5.2.8) (11). Raw files of the different experiments were processed separately. The derived peak list was searched using Andromeda search engine integrated in MaxQuant (12) against a reference *E. coli* K-12 proteome (taxonomy ID 83333) obtained from UniProt (4403 protein entries, released in October 2020), and a file containing 245 common laboratory contaminants. During the first search, peptide mass tolerance was set to 20 ppm (parts per million) and, in the main search, to 4.5 ppm. For triple-label SILAC experiments, multiplicity was set to three with Lys4 and Lys8 specified as medium and heavy labels, respectively. Methionine oxidation, protein N-terminal acetylation, and Ser-Thr-Tyr phosphorylation were defined as variable modifications, and carbamidomethylation of cysteines was set as a fixed modification. The minimum required peptide length was set to seven amino acids with the maximum of two missed cleavages allowed for endoproteinase Lys-C that was set to specifically cleave at lysine C terminus. Chymotrypsin was set to specifically cleave at phenylalanine, tryptophan, tyrosine, leucine, and methionine C terminus with maximum five missed cleavages allowing for maximum of four labeled amino acids. To increase the number of quantified features, the “match between runs” option was enabled with a match time window set to 0.7 min. Re-quantify option was

enabled to allow for quantification of SILAC and dimethyl-labelled pairs that result in extreme ratio values. All (phospho)peptide and protein identifications were filtered using a target-decoy approach with a false discovery rate (FDR) set to 0.01 at peptide and protein level (13). Proteins identified by the same set of peptides were combined to a single protein group. Protein groups identified by a single peptide were kept in the data set. For protein quantification, a minimum of two peptide ratio counts was required. To increase the number of quantified features, the “match between runs” option was enabled with a match time window set to 0.7 min. This allows the transfer of peptide identifications across LC-MS/MS runs based on the mass and the retention time of the peptide identified by MS/MS. Re-quantify option was enabled to allow for quantification of SILAC pairs that result in extreme ratio values. For proteome dynamic analysis under low YjjJ induction and *in vitro* kinase assay, MaxQuant settings were slightly changed: for double dimethyl-labelling, multiplicity was set to two (DimethylLys0 and DimethylLys4), no phosphorylation was defined as variable modification and maximum missed cleavage for trypsin was set to 2; for *in vitro* kinase experiment, no multiplicity was defined. Statistical analysis of MaxQuant output data was performed by using Perseus software (version 1.6.5.0) (14), and figures were edited in Adobe Illustrator. All contaminants and reverse hits were removed. Phosphorylation sites were additionally filtered for posterior error probability scores of <0.01. Minimal score of 40 was required for phosphorylation site and 20 for protein identifications. Changes in phosphorylation events were normalized to differences in protein abundances, unless otherwise stated. For that, phosphorylation site SILAC ratios were divided with the protein SILAC ratios of corresponding proteins. Normalized phosphorylation site ratios were log2-transformed and plotted against the log10-transformed phosphopeptide intensities summed for each of two SILAC channels observed. Statistically significantly regulated phosphorylation

sites were determined by applying an arbitrary ratio threshold of 2 in log2 scale (fourfold). Statistically significantly regulated proteins were determined by using significance B test with a P value of 0.05. For Volcano plots, log2-transformed ratios of three independent experiments were grouped into one group and compared to the group containing only zero values using t test with FDR of 0.1 and the minimal fold change S0 of 0. Based on majority protein ID, identified hits were annotated (GOBP, GOMF, GOCC and Reactome). Dataset was then filtered based on t-test significance >1, Fisher exact test was performed (FDR of 0.1) and the generated matrix was visualized with in-house software. For dynamic-proteome analysis, proteins present in at least 2 replicates in one time point were selected and an analyzed via ANOVA (FDR of 0.1 and the minimal fold change S0 of 0). On significant proteins, Post-hoc test was performed (FDR of 0.1) and results plot as heat map. As for SILAC-based experiment, proteins were then annotated, followed by Fisher exact test and then visualized.

Protein purification

For CsrA, CsrA S56,59E and YjjJ purifications, IPTG-induced *E. coli* One Shot BL21 (DE3) containing the respective plasmid (pET28-CsrA, pET28-CsrA S56,59E, pET28-YjjJ) were cultured in LB Medium at 37 °C. Protein expression was induced at OD_{600} = 0.8. After 3 hours at 37°C, bacteria were harvested by centrifugation and lysed by sonication (30 s, 50 % power, five times) in HisTrap buffer A (50 mM Tris-HCl pH 7.8, 1 M NaCl, 1 M Urea, 5 mM $MgSO_4$, 5 mM β-mercaptoethanol, 5 % glycerol, 5 mM imidazole, 1 tablet per 500 ml complete EDTA-free protease inhibitor cocktail (Roche)). The lysate was clarified by centrifugation (37,500 g, 30 min, 4 °C) and the supernatant was applied to a 1 mL Ni-NTA HisTrap column (GE Healthcare). The protein was eluted using HisTrap buffer B (HisTrap buffer A with 300 mM

imidazole) and analyzed by SDS–PAGE. Further protein purification was achieved by size exclusion chromatography (SEC) through a SuperdexTM 200 10/300 GL column (GE Healthcare) using a buffer containing 50 mM Tris pH = 7.5, 1 M NaCl. Fractions of interest were analyzed by SDS–PAGE, pooled and concentrated in Amicon Ultra-4 centrifugal filters (MWCO 3 or 10 kDa, centrifugation at 2,000 rpm, 4°C). Protein concentration was measured with the NanoDrop ND-1000 Spectrophotometer. Proteins were finally stored in buffer supplemented with 50 % glycerol at -20 °C. For RpmE purification, cells were cultured overnight (16-18 hours) in LB supplemented with 20% lactose for induction. Cells were pellet by centrifugation and lysed in BufferAHepes A (20 mM Hepes, 1mM $NaCl_2$, 20 mM $MgCl_2$, 20mM KCl, 5 mM β-mercaptoethanol, 5 % glycerol, 50 mM imidazole, 1 tablet per 500 ml complete EDTA-free protease inhibitor cocktail (Roche)). The lysate was clarified by ultracentrifugation (50,500 g, 45 min, 4 °C) and the supernatant was mixed to a 1 ml Protino Ni-NTA slurry (Macherey/Nagel) and load into an empty column. The protein was eluted using BufferAHepes B (BufferAHepes with 250 mM imidazole). Sample was concentrated with Amicon Ultra-4 centrifugal filter (MWCO 3 kDa, 4000 rpm, 4°C) and one volume of glycerol was added. Buffer was exchanged to SECHepes (20 mM Hepes, 1mM $NaCl_2$, 20 mM $MgCl_2$, 20mM KCl, 5 mM β-mercaptoethanol, 50 % glycerol) using same centrifugal filter and stored at -20°.

Ribosome purification and density gradient

E. coli MG1655 harbouring the empty vector (pBAD33), the *yjjJ*-expression plasmid and the plasmid carrying the mutant variant of *yjjJ S342,364Q* ($yjjJ^{DK}$) were cultured in 20 ml LB supplemented with chloramphenicol over night at 37°C. 100 ml of LB without the addition of

chloramphenicol were then inoculated at OD_{578} 0.08 and incubated at 37°C in an aerial shaker (200 rpm). Induction of *yjjJ* and *yjjJ*^{DK} expression was induced by adding 0.01% arabinose, when the cultures reached OD_{578} 0.3. Cultures were harvested by centrifugation (5 min, 3800 rpm, 4°C) when they reached OD_{578} 0.7. Pellets were subsequently resuspended in 1 ml ribosome buffer (RB; 25 mM HEPES pH7.5, 30 mM MgOAc, 150 mM KOAc, 50 µg/ml chloramphenicol, 0.025% DDM (dodecyl-β-D-maltoside) and drops were frozen in liquid nitrogen. Frozen samples were grinded under cryogenic conditions using a CryoMill (Retsch GmbH, Haan, Germany) and dissolved in 10 ml RB. These lysates were layered on top of 12.5 ml 32% sucrose cushions prepared in RB and centrifuged for 18 h at 100.000xg in a Ti70 rotor (Beckman Coulter, Brea, USA), respectively. Clear ribosome pellets were dissolved in 200 µl RB and measured for their absorption at 260 nm using a NanoDrop (ThermoFisher, Waltham, USA). 25 µl of A_{260} = 40 were loaded on top of a 10-40% sucrose gradient (14 ml in RB) prepared with GradientMaster (Biocomp, Fredericton, Canada) and centrifuged in a SW40 rotor (Beckman Coulter, Brea, USA) at 200.000xg, 4°C for 5 h. Sucrose density gradients were analysed and fractionated using a Piston Gradient Fractionator (Biocomp, Fredericton, Canada) and a Triax Flow Cell model FC-1 for 260 nm scans (Biocomp, Fredericton, Canada). Fractions were immediately snap frozen in liquid nitrogen and stored at -80°C.

Transduction of *rpmE* and *yjjJ* deletion

For the P1*vir* lysate, the KEIO donor strains were grown overnight in LB at 37°. The next day the overnight culture is diluted 1:100 in 5 ml of LB supplemented with 0.2% glucose and 5 mM $CaCl_2$ and incubated for 30 minutes, followed by addition of 0.1ml P1*vir* lysate. The culture is incubated for 2-3 hours, till cellular lysis, and then add 0.1 ml Chloroform and vortex.

Culture is then centrifuged (4500 rpm for 10 minutes), and sterile filtered (0.2 µm). For transduction, 1-2 colonies of recipient strain, MG1655 were incubated in the morning in 5ml of LB with 0.2% glucose and 5 mM $CaCl_2$. When culture reached an OD_{600} of 0.8-1, it was harvested by centrifugation (5000 rpm for 5 min) and then resuspended in 1 ml LB supplemented with 0.5 mM $CaCl_2$ and 100 mM $MgSO_4$. Resuspended pellet was split in different tubes (100 µl each) and incubated with different dilution of P1*vir* lysate and incubated at 37°C without shaking. As control, 100 µl of culture and 100 µl of P1*vir* lysate were supplemented with sterile LB. To stop phage infection, 200 µl of 1M Sodium Citrate was added to each sample followed incubation at 37°C with shaking. After 1 hour, cultures were pellet (1 min, 13000 rpm), resuspended in 100 µl LB with 100 mM Sodium citrate and plated on LB supplemented with 50 µg/ml kanamycin and 20 mM sodium citrate. Plate were incubated overnight and single colonies were streaked repeatedly on fresh plates for several days, until no further phage lysis can be observed. Resistant colonies were checked for correct deletion via PCR and sequencing.

CRISPRi-Silencing of *yjjJ*

CRISPRi repression assays were performed with the *E. coli* strain *YYdCas9:BW25993 intC::TetR-dcas9-aadA laqY::ypet-cat* (5) which carries a chromosomal expression cassette for dcas9 and tetR (8) under the control of an anhydrotetracyclin (aTc) -inducible promoter. *yjjJ*-specific sgRNA was cloned into plasmid pgRNA-bacteria (8) by inverse PCR using yjjJ_Ec-F-fwd and Ec-F-rev primers. The cloned spacer sequence in primer yjjJ_Ec-F-fwd, 5'-CGCAATTCCGGGGCAGAACG-3' is complementary to nucleotide positions 37-52 of the *yjjJ* gene. Transformation of *YYdCas9:BW25993* with the *yjjJ*-pgRNA plasmid results in

constitutive sgRNA expression whereas dCas9 expression can be induced by the addition of 100 ng/ml aTc to the medium.

Determination of growth rate and RNA preparation

To investigate the growth of *YYdCas9:BW25993 intC::TetR-dcas9-aadA laqY::ypet-cat* bacteria transformed with the *yjjJ*-pgRNA plasmid under not induced (normal) and induced (dCas9 active to silence *yjjJ*) conditions, we performed growth assays (OD_{600}) with monoclonal cultures in M9 minimal medium by using a Tecan Infinite 200 Pro plate reader. At the endpoint of three independent growth assays (20hrs), bacteria were pelleted by centrifugation and total RNA was isolated with the TRIzol reagent. The isolated RNAs were treated with DNAse I to remove any contaminating genomic DNA. Subsequently all RNAs were further purified and concentrated with Qiagen RNeasy spin columns. First strand cDNA synthesis was performed with the LunaScript RT Supermix Kit (New England Biolabs) by using 200 ng RNA for each cDNA synthesis.

Real-time PCR

Real-time PCR was performed using FastStart Universal SYBR Green Master Mix (Roche) with cDNA derived from three independent growth assays under normal and inductive (aTc) conditions. To normalize the amount of cDNA between samples, expression of the endogenous control gene, *hcaT* was analyzed alongside with *yjjJ* expression. Additionally, no template controls were prepared for each primer pair to control for cross-contaminations. Each combination of primer pair and cDNA was measured in triplicates of 10 µl from a 35 µl master.

References:

1. Semanjski M, Germain E, Bratl K, Kiessling A, Gerdes K, Macek B. 2018. The kinases HipA and HipA7 phosphorylate different substrate pools in Escherichia coli to promote multidrug tolerance. Science Signaling 11:5750.
2. Gibson DG, Young L, Chuang RY, Venter JC, Hutchison CA, Smith HO. 2009. Enzymatic assembly of DNA molecules up to several hundred kilobases. Nature Methods 6:343-345.
3. Baba T, Ara T, Hasegawa M, Takai Y, Okumura Y, Baba M, Datsenko KA, Tomita M, Wanner BL, Mori H. 2006. Construction of Escherichia coli K-12 in-frame, single-gene knockout mutants: The Keio collection, vol 2.
4. Germain E, Castro-Roa D, Zenkin N, Gerdes K. 2013. Molecular Mechanism of Bacterial Persistence by HipA. Molecular Cell 52:248-254.
5. Lawson MJ, Camsund D, Larsson J, Baltekin O, Fange D, Elf J. 2017. In situ genotyping of a pooled strain library after characterizing complex phenotypes. Mol Syst Biol 13:947.
6. Guzman LM, Belin D, Carson MJ, Beckwith J. 1995. Tight regulation, modulation, and high-level expression by vectors containing the arabinose PBAD promoter, vol 177, p 4121-4130.
7. Gotfredsen M, Gerdes K. 1998. The Escherichia coli relBE genes belong to a new toxin–antitoxin gene family, vol 29, p 1065-1076.
8. Qi LS, Larson MH, Gilbert LA, Doudna JA, Weissman JS, Arkin AP, Lim WA. 2013. Repurposing CRISPR as an RNA-guided platform for sequence-specific control of gene expression. Cell 152:1173-83.
9. Soufi B, Macek B. 2014. Stable Isotope Labeling by Amino Acids Applied to Bacterial Cell Culture. doi:10.1007/978-1-4939-1142-4_2:9-22.

10. Rappsilber J, Mann M, Ishihama Y. 2007. Protocol for micro-purification, enrichment, pre-fractionation and storage of peptides for proteomics using StageTips. Nature Protocols 2:1896-1906.

11. Cox J, Mann M. 2008. MaxQuant enables high peptide identification rates, individualized p.p.b.-range mass accuracies and proteome-wide protein quantification. Nature Biotechnology 26:1367-1372.

12. Cox J, Neuhauser N, Michalski A, Scheltema RA, Olsen JV, Mann M. 2011. Andromeda: A peptide search engine integrated into the MaxQuant environment. Journal of Proteome Research 10:1794-1805.

13. Elias JE, Gygi SP. 2007. Target-decoy search strategy for increased confidence in large-scale protein identifications by mass spectrometry. Nature Methods 4:207-214.

14. Tyanova S, Temu T, Sinitcyn P, Carlson A, Hein MY, Geiger T, Mann M, Cox J. 2016. The Perseus computational platform for comprehensive analysis of (prote)omics data. Nature Methods 13:731-740.
